# Racial and Ethnic Disparities in Geographic Access to Autism Resources Across the US

**DOI:** 10.1001/jamanetworkopen.2022.51182

**Published:** 2023-01-23

**Authors:** Bennett M. Liu, Kelley Paskov, Jack Kent, Maya McNealis, Soren Sutaria, Olivia Dods, Christopher Harjadi, Nate Stockham, Andrey Ostrovsky, Dennis P. Wall

**Affiliations:** 1 Department of Pediatrics, Division of Systems Medicine, Stanford University, Stanford, California; 2 Social Innovation Ventures, Lewes, Delaware

## Abstract

**Importance:**

While research has identified racial and ethnic disparities in access to autism services, the size, extent, and specific locations of these access gaps have not yet been characterized on a national scale. Mapping comprehensive national listings of autism health care services together with the prevalence of autistic children of various races and ethnicities and evaluating geographic regions defined by localized commuting patterns may help to identify areas within the US where families who belong to minoritized racial and ethnic groups have disproportionally lower access to services.

**Objective:**

To evaluate differences in access to autism health care services among autistic children of various races and ethnicities within precisely defined geographic regions encompassing all serviceable areas within the US.

**Design, Setting, and Participants:**

This population-based cross-sectional study was conducted from October 5, 2021, to June 3, 2022, and involved 530 965 autistic children in kindergarten through grade 12. Core-based statistical areas (CBSAs; defined as areas containing a city and its surrounding commuter region), the Civil Rights Data Collection (CRDC) data set, and 51 071 autism resources (collected from October 1, 2015, to December 18, 2022) geographically distributed into 912 CBSAs were combined and analyzed to understand variation in access to autism health care services among autistic children of different races and ethnicities. Six racial and ethnic categories (American Indian or Alaska Native, Asian, Black or African American, Hispanic or Latino, Native Hawaiian or other Pacific Islander, and White) assigned by the US Department of Education were included in the analysis.

**Main Outcomes and Measures:**

A regularized least-squares regression analysis was used to measure differences in nationwide resource allocation between racial and ethnic groups. The number of autism resources allocated per autistic child was estimated based on the child’s racial and ethnic group. To evaluate how the CBSA population size may have altered the results, the least-squares regression analysis was run on CBSAs divided into metropolitan (>50 000 inhabitants) and micropolitan (10 000-50 000 inhabitants) groups. A Mann-Whitney *U* test was used to compare the model estimated ratio of autism resources to autistic children among specific racial and ethnic groups comprising the proportions of autistic children in each CBSA.

**Results:**

Among 530 965 autistic children aged 5 to 18 years, 83.9% were male and 16.1% were female; 0.7% of children were American Indian or Alaska Native, 5.9% were Asian, 14.3% were Black or African American, 22.9% were Hispanic or Latino, 0.2% were Native Hawaiian or other Pacific Islander, 51.7% were White, and 4.2% were of 2 or more races and/or ethnicities. At a national scale, American Indian or Alaska Native autistic children (β = 0; 95% CI, 0-0; *P* = .01) and Hispanic autistic children (β = 0.02; 95% CI, 0-0.06; *P* = .02) had significant disparities in access to autism resources in comparison with White autistic children. When evaluating the proportion of autistic children in each racial and ethnic group, areas in which Black autistic children (>50% of the population: β = 0.05; <50% of the population: β = 0.07; *P* = .002) or Hispanic autistic children (>50% of the population: β = 0.04; <50% of the population: β = 0.07; *P* < .001) comprised greater than 50% of the total population of autistic children had significantly fewer resources than areas in which Black or Hispanic autistic children comprised less than 50% of the total population. Comparing metropolitan vs micropolitan CBSAs revealed that in micropolitan CBSAs, Black autistic children (β = 0; 95% CI, 0-0; *P* < .001) and Hispanic autistic children (β = 0; 95% CI, 0-0.02; *P* < .001) had the greatest disparities in access to autism resources compared with White autistic children. In metropolitan CBSAs, American Indian or Alaska Native autistic children (β = 0; 95% CI, 0-0; *P* = .005) and Hispanic autistic children (β = 0.01; 95% CI, 0-0.06; *P* = .02) had the greatest disparities compared with White autistic children.

**Conclusions and Relevance:**

In this study, autistic children from several minoritized racial and ethnic groups, including Black and Hispanic autistic children, had access to significantly fewer autism resources than White autistic children in the US. This study pinpointed the specific geographic regions with the greatest disparities, where increases in the number and types of treatment options are warranted. These findings suggest that a prioritized response strategy to address these racial and ethnic disparities is needed.

## Key Points

**Question:**

Do autistic children belonging to minoritized racial and ethnic groups have access to fewer autism resources than White autistic children in the US and, if so, where are these disparities most significant?

**Findings:**

In this cross-sectional study involving 530 965 autistic children and 51 071 autism services in the US, analyses by core-based statistical area revealed that American Indian or Alaska Native, Black or African American, and Hispanic or Latino autistic children had access to significantly fewer resources than White autistic children.

**Meaning:**

These findings suggest that autistic children from minoritized racial and ethnic groups experience significant disparities in access to autism services, with certain core-based statistical areas having greater inequities than others, necessitating a prioritized response strategy to address these disparities.

## Teaser

This cross-sectional study assesses racial and ethnic disparities in access to autism resources among autistic children across geographic regions in the US.

## Introduction

Autism spectrum disorder (ASD) is a growing pediatric health concern in the US. Over the past 5 years, its prevalence has tripled from 1 in 125 children to 1 in 44.^1,2^ While autism is a highly heterogeneous condition, it often results in repetitive and restrictive behaviors, language challenges, and social interaction deficits.^3,4^ Early diagnosis and behavioral intervention are key components of improving behavior, social communication, and emotion recognition in autistic children.^4,5,6^

In the past 3 decades, researchers and advocacy groups have identified a pattern of racial disparities in access to health care services.^7,8,9,10,11^ While social factors associated with health, such as an individual’s economic stability^12^ and community context,^13^ have been associated with inequities in access to health care services for minoritized racial and ethnic populations, these gaps in access to care occur primarily because of the inadequacy of larger systems.^14,15^ Patients from minoritized racial and ethnic groups seeking medical attention for conditions such as cardiovascular disease,^16,17^ cancer,^18,19^and diabetes^20,21^ may receive substandard care in comparison with White patients.

Within the field of autism research, there has been a movement to examine the efficacy of evidence-based practices through implementation science.^22,23,24,25^ With regard to racial and ethnic differences in the implementation of autism treatment, studies have highlighted disparities that exist for minoritized racial and ethnic groups in the early identification of autism as well as the differential use patterns of ASD resources through school systems, academic hospitals, and insurance billing codes.^26,27,28,29,30,31,32,33,34,35,36,37^ However, the research community has yet to characterize the extent to which geographic proximity to autism resources varies between racial and ethnic groups on a national scale. The Autism and Developmental Disabilities Monitoring (ADDM) Network of the Centers for Disease Control and Prevention reports diagnostic differences by racial and ethnic group annually to track inequity in 11 states in the US.^2^ While the ADDM Network report has been an influential step in understanding the locations where autism resource disparities are most prevalent for minoritized racial and ethnic populations, there is a lack of information regarding the 39 states not captured in the ADDM Network analysis. In addition, a national analysis subdivided by state cannot provide a granularized report of where these inequities exist, which may be due to the limited amount of research on autism treatment disparities and the broader challenge of obtaining high-quality disparity measurements.^38^

We addressed the question of how geography intersects with racial disparities in autism treatment using geographic boundaries defined by localized commuting patterns to identify areas within the US where families from minoritized racial and ethnic groups have disproportionally lower access to services. To do so, we used an online resource tracking and mapping tool, GapMap, as well as data from the Civil Rights Data Collection (CRDC) survey. The GapMap data set contains 51 071 diagnostic and treatment resources for autism, and the CRDC reports on autism prevalence from every public school (kindergarten through grade 12) in the US, including 534 847 autistic children. The goal of this study was not only to highlight inequities in access to autism services, but to improve opportunities for all families of autistic children.

## Methods

This population-based cross-sectional study was conducted from October 5, 2021, to June 3, 2022, and involved 530 965 US autistic children receiving autism services in their schools during the 2017 to 2018 school year (using data published in 2020). Data were obtained using the GapMap database and the CRDC survey and represented information from 912 core-based statistical areas (CBSAs), which are geographic divisions of the US based on commuting times to urban centers. Because all data used were deidentified, this study was not considered human participant research by Stanford University and was deemed exempt from institutional review board approval and informed consent. This study followed the Strengthening the Reporting of Observational Studies in Epidemiology (STROBE) reporting guideline for cross-sectional studies.

### Autism Prevalence: the CRDC Data Set

The CRDC is a biennial survey of public schools that has been required by the Office of Civil Rights of the US Department of Education since 1968. The survey collects data from all public schools and educational agencies, including juvenile justice facilities, charter schools, alternative schools, and schools serving only children with disabilities (with a response rate of 99.8%^39^). We used the most recent version of the CRDC, which was published in 2020 and reflected the collection years of 2017 to 2018. This version contained enrollment data on 50.9 million children in kindergarten through grade 12 from 97 632 schools in all 50 states plus Puerto Rico.^40^

The CRDC data set contains the number of children receiving special education services for ASD under the Individuals with Disabilities Education Act and is organized by CBSA.^41^ Overall, 90% of children receiving autism services in school exceeded the threshold for autism based on criteria from the Autism Diagnostic Observation Schedule (first and second editions).^42^ An estimated 85.1% to 93.6% of children with an autism diagnosis are receiving autism services in school.^43^ Unlike the medical system, nearly all children in the country interact with schools regularly, regardless of their race and ethnicity. This routine interaction makes the CRDC a reliable source for estimating the prevalence of school-aged children with ASD. The CRDC divides individual counts of children receiving special education services for ASD into 6 racial and ethnic categories: American Indian or Alaska Native, Asian, Black or African American (hereinafter referred to as Black), Hispanic or Latino (hereinafter referred to as Hispanic), Native Hawaiian or other Pacific Islander, and White (eAppendix 1 in Supplement 1). Data on self-reported race and ethnicity were gathered and verified by the school districts.^44^ The racial and ethnic categories were defined by the US Department of Education for the 2017 to 2018 collection period.^45^ The final data set included 530 965 autistic children from 97 632 schools; 37 CBSAs (in Iowa, Puerto Rico, and Vermont) were removed due to data issues, for a total of 3882 children and 428 autism resources excluded from the analysis.

### Autism Resources: GapMap Data Set

**Figure 1.**
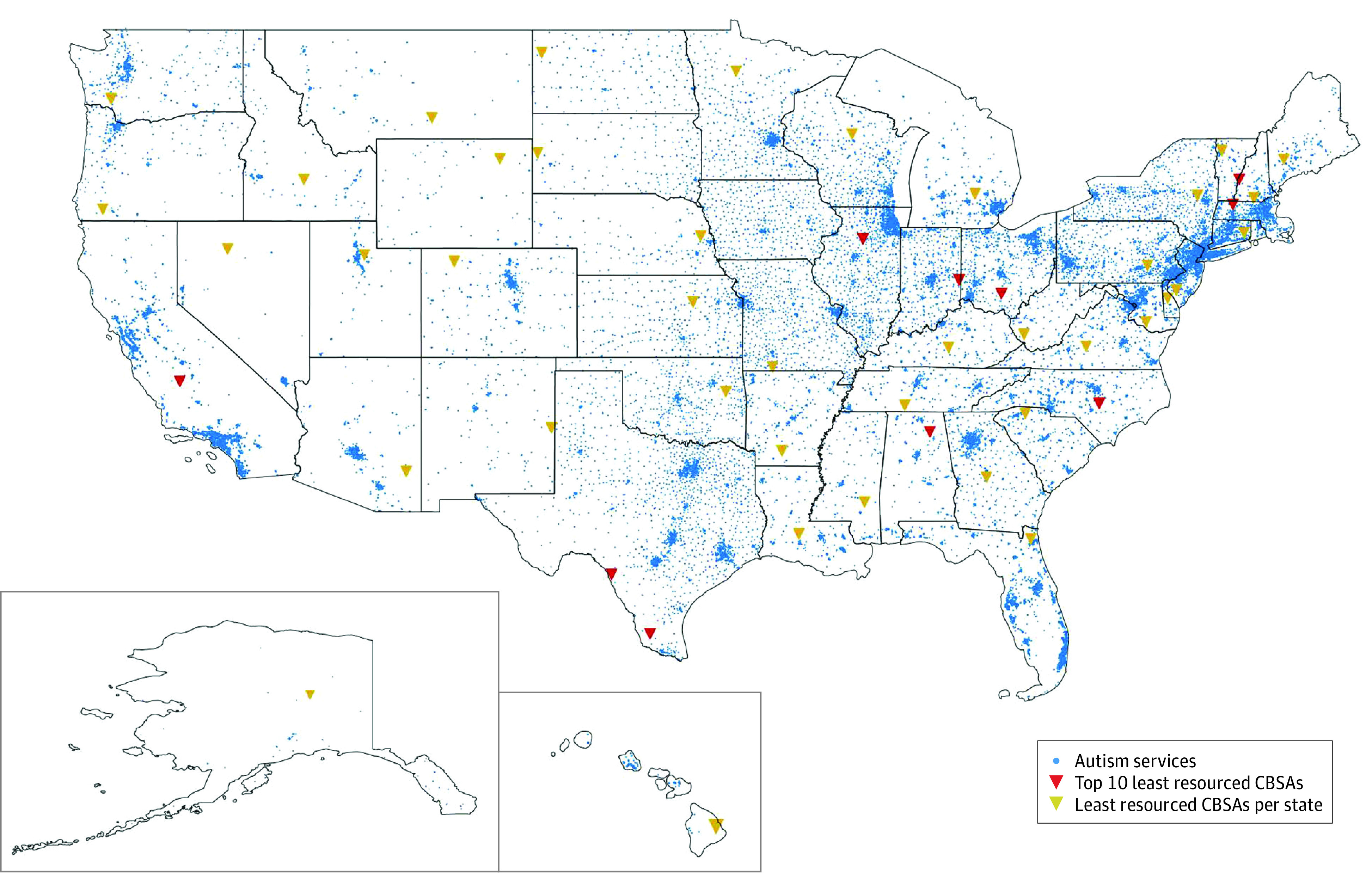
Autism Resources and Top 10 Underresourced Core-Based Statistical Areas (CBSAs) Full details on resource availability for all CBSAs in the US are available in eAppendix 3 in Supplement 1.

The GapMap data set^46^ contains more than 51 071 autism resources in the US (Figure 1). Resources were compiled from Autism Speaks,^47^ Autism Source,^48^ Parents Helping Parents,^49^ and Google Places^50^ from October 1, 2015, to December 18, 2022. After collection, resources were deduplicated and verified using manual curation and the Google Places application programming interface.^51^ Resource service categories included diagnostic services, therapies, services for adults with ASD, school resources, and other services related to autism (eg, health, education, and recreation) (eAppendix 2 in Supplement 1).

### Geographic Areas: CBSAs

While statewide analysis provides a high-level description of geographic disparities in resource prevalence, we sought a more granular analysis. We used CBSAs determined by the Office of Management and Budget^52^ to compare resource distribution and access disparities in the US. A CBSA is a geographic entity made up of a county or counties containing a population nucleus and adjacent areas that have socioeconomic and commuter-based integration with that nucleus. CBSAs are divided into metropolitan statistical areas (>50 000 inhabitants) and micropolitan statistical areas (10 000-50 000 inhabitants).^53^ Approximately 86.3% of the population resides in metropolitan CBSAs, 8.3% resides in micropolitan CBSAs, and 5.4% resides in rural areas not included in our analysis. The Office of Management and Budget establishes and maintains these areas for statistical purposes.^52^ Due to the focus on functional commuting-based grouping and the high degree of standardization and adoption of these geographic delineations, CBSAs are a valuable tool for understanding a population’s access to services.

### Statistical Analysis

To understand the differences in access to ASD resources between the 6 racial and ethnic groups, we used a linear least-squares regression analysis with a nonnegativity constraint to model the number of GapMap autism resources within each CBSA as a function of the racial and ethnic composition of autistic children in a CBSA. Our model produced a nationwide estimate of the number of resources available per child depending on the child’s race and ethnicity. *P* values and 95% CIs were bootstrapped with 1000 replicates drawn at the level of CBSA. *P* values were then calculated by evaluating whether children within each minoritized racial and ethnic group had access to fewer autism resources than White children (the reference group).

We first performed an analysis of all CBSAs to understand how the different racial and ethnic groups compared in their access to resources at a national level. Next, due to different population sizes, geographic spread, and resource access between metropolitan and micropolitan areas, we divided CBSAs into metropolitan and micropolitan groups and reran our model to understand how the CBSA population size may have altered the model’s results.

We then combined the number of resources and the number of autism cases for CBSAs with greater than 50% autism prevalence and separately for CBSAs with fewer than 50% of autistic children belonging to the same racial and ethnic group. We then calculated the ratio of total autism resources to total autistic children. We used the Mann-Whitney *U* test to compare the ratio of autism resources to autistic children among specific racial and ethnic groups comprising the proportion of autistic children in each CBSA) to account for the potential nonnormality. American Indian or Alaska Native, Asian, and Native Hawaiian or other Pacific Islander children were excluded from this analysis because there were fewer than 10 CBSAs in which these racial and ethnic groups comprised the largest proportion.

Data were analyzed using Python in Jupyter Notebooks software, version 6.3.0 (Jupyter). The threshold for statistical significance was 2-sided *P* = .05.

## Results

All states contained at least 1 CBSA with disproportionately low access to autism health care resources (Figure 1). A total of 84 CBSAs in our data set had no autism resources. Among 530 965 children aged 5 to 18 years with a confirmed autism diagnosis, 445 273 (83.9%) were male and 85 692 (16.1%) were female. With regard to racial and ethnic categories, 3738 children (0.7%; 3157 male and 581 female) were American Indian or Alaska Native, 31 284 (5.9%; 25 905 male and 5379 female) were Asian, 75 769 (14.3%; 63 583 male and 12 186 female) were Black, 121 525 (22.9%; 103 208 male and 18 317 female) were Hispanic, 1320 (0.2%; 1093 male and 227 female) were Native Hawaiian or other Pacific Islander, 274 769 (51.7%; 229 702 male and 45 067 female) were White, and 22 560 (4.2%; 18 625 male and 3935 female) were of 2 or more races and/or ethnicities.

**Figure 2.**
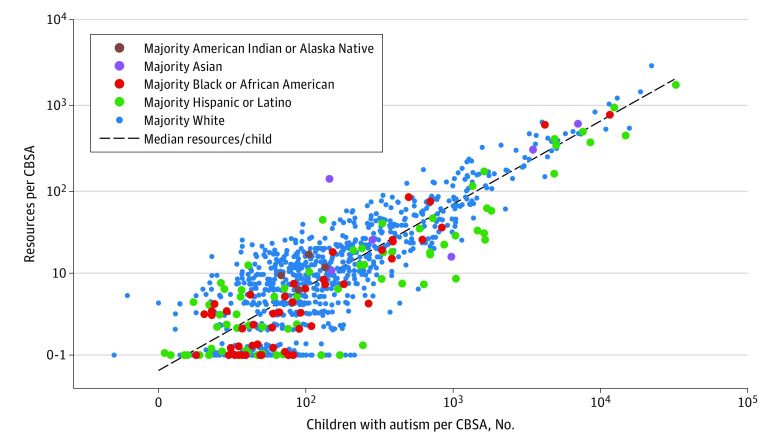
Comparison Between Autism Resources and Autistic Children Within Each Core-Based Statistical Area (CBSA)

Comparing the total number of autistic children with the raw total number of autism resources per CBSA revealed that several minoritized racial and ethnic groups had access to fewer than the median number of resources per autistic child (Figure 2). Our 3 analyses suggested that CBSAs with higher counts of Black or Hispanic autistic children had fewer resources per child than the median number of CBSA resources per child. For example, the autistic population in the Rio Grande City–Roma (Texas) CBSA was 99.4% Hispanic, and this CBSA had 0.006 resources per child compared with a median of 0.070 resources per child for all CBSAs (>10 times fewer resources than the median) (eAppendix 3 in Supplement 1). Seven of the 15 most underresourced CBSAs were in southern states (Alabama, Mississippi, North Carolina, and Texas), and 4 CBSAs (in California and Texas) had a majority Hispanic population (Table).

**Table.** The 15 Core-Based Statistical Areas With the Fewest Autism Resources per Child^a^

CBSA searchable address	CBSA title	CBSA state	CBSA classification	Resources per child^b^	Total autistic children, No.	Autistic children by race and ethnicity, No. (%)^c^					
						American Indian or Alaska Native	Asian	Black or African American	Hispanic or Latino	Native Hawaiian or other Pacific Islander	White
17200	Claremont-Lebanon	New Hampshire and Vermont	Micropolitan	0	213	1 (0.5)	1 (0.5)	4 (1.9)	2 (0.9)	0	202 (94.8)
36860	Ottawa-Peru	Illinois	Micropolitan	0	200	0	3 (1.5)	1 (0.5)	26 (13.0)	0	163 (81.5)
20380	Dunn	North Carolina	Micropolitan	0	186	1 (0.5)	0	45 (24.2)	31 (16.7)	0	99 (53.2)
40100	Rio Grande City	Texas	Micropolitan	0	170	0	0	0	169 (99.4)	0	1 (0.6)
10700	Albertville	Alabama	Micropolitan	0	143	0	0	3 (2.1)	18 (12.6)	0	120 (83.9)
17060	Chillicothe	Ohio	Micropolitan	0	143	0	0	3 (2.1)	1 (0.7)	0	132 (92.3)
24640	Greenfield Town	Massachusetts	Micropolitan	0	136	0	1 (0.7)	1 (0.7)	10 (7.4)	0	113 (83.1)
20580	Eagle Pass	Texas	Micropolitan	0	127	3 (2.4)	0	0	123 (96.9)	0	1 (0.8)
39980	Richmond	Indiana	Micropolitan	0	126	0	0	8 (6.3)	7 (5.6)	0	101 (80.2)
25260	Hanford-Corcoran	California	Metropolitan	0.004	243	2 (0.8)	8 (3.3)	17 (7.0)	124 (51.0)	0	87 (35.8)
15180	Brownsville-Harlingen	Texas	Metropolitan	0.008	1041	1 (0.1)	3 (0.3)	6 (0.6)	994 (95.5)	0	33 (3.2)
41820	Sanford	North Carolina	Micropolitan	0	110	1 (0.9)	2 (1.8)	17 (15.5)	32 (29.1)	0	53 (48.2)
29860	Laurel	Mississippi	Micropolitan	0	108	0	0	37 (34.3)	3 (2.8)	0	68 (63.0)
27920	Junction City	Kansas	Micropolitan	0	100	0	0	21 (21.0)	15 (15.0)	0	54 (54.0)
37020	Owosso	Michigan	Micropolitan	0	100	0	1 (1.0)	0	1 (1.0)	0	90 (90.0)

Abbreviation: CBSA, core-based statistical area.

^a^ A total of 84 CBSAs in our data set had no autism resources, so we chose to show the underresourced CBSAs that had the largest populations of autistic children. The full data set is provided in eAppendix 3 in Supplement 1.

^b^ Ratio of total number of autism resources to total number of autistic children.

^c^ Percentages were calculated across rows (based on the total number of autistic children in the respective CBSA).

### National Analysis

The resource allocation regression model revealed that compared with White children, American Indian or Alaska Native children (β = 0; 95% CI, 0-0; *P* = .01) and Native Hawaiian or other Pacific Islander children (β = 0; 95% CI, 0-2.07; *P* = .28) had access to the fewest autism resources per child, followed by Hispanic children (β = 0.02; 95% CI, 0-0.06; *P* = .02) and Black children (β = 0.06; 95% CI, 0-0.13; *P* = .24) (eFigure in Supplement 1). Asian children (β = 0.16; 95% CI, 0.01-0.32; *P* = .79) had access to the most resources per child compared with White children.

### Metropolitan and Micropolitan CBSA Analysis

**Figure 3.**
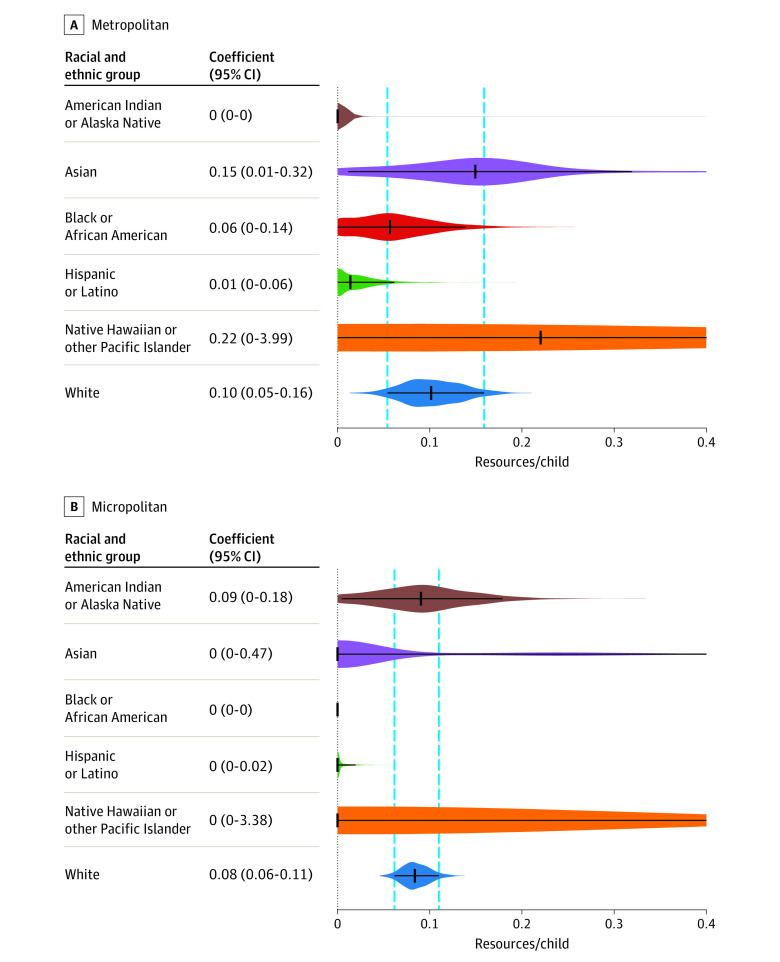
Metropolitan vs Micropolitan Differences in Access to Autism Resources by Racial and Ethnic Group Each plot shows the bootstrapped distributions of coefficients for each group. Black solid vertical lines represent the coefficient. Black horizontal lines represent bootstrapped 95% CIs. The linear regression with the constraint of nonnegative coefficients makes it such that estimates stemming from racial and ethnic populations with small numbers in the US (eg, American Indian or Alaska Native) could have large CIs and/or resolve to low estimates or even zero in the model. Vertical dotted lines show 95% CIs for the reference group (White children).

Across micropolitan CBSAs, compared with White children, Black children had access to the fewest autism resources per child (β = 0; 95% CI, 0-0; *P* < .001), followed by Hispanic children (β = 0; 95% CI, 0-0.02; *P* < .001) (Figure 3B). For the analysis of metropolitan CBSAs, the model revealed that American Indian or Alaska Native children (β = 0; 95% CI, 0-0; *P* = .005) and Hispanic children (β = 0.01; 95% CI, 0-0.06; *P* = .02) had access to the fewest resources per child compared with White children (Figure 3A).

### Analysis by Proportion of Autistic Children in Racial and Ethnic Groups in Each CBSA

**Figure 4.**
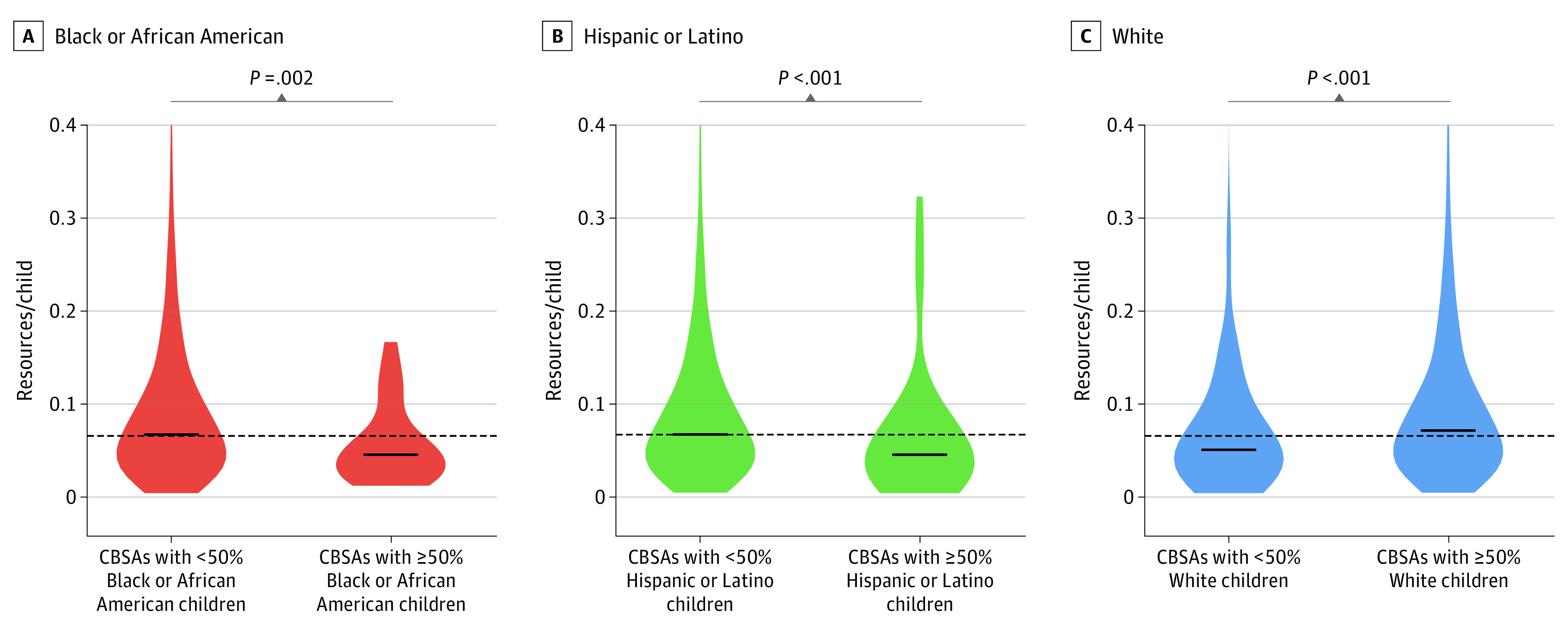
Differences in Access to Autism Resources by Proportion of Autistic Children in Each Racial and Ethnic Group in Each Core-Based Statistical Areas (CBSAs) The solid black lines indicate the medians of the racial and ethnic group, the black dashed line represents the median of the total population, and the colored areas are the bootstrap distributions. *P* values were calculated using a Mann-Whitney *U* test.

The CBSAs in which Black children comprised greater than 50% of the total population of autistic children had significantly fewer resources per child than CBSAs in which Black children comprised less than 50% of the total population of autistic children (>50% of the population: β = 0.05; <50% of the population: β = 0.07; *P* = .002). In addition, there were significantly fewer resources per child in areas in which Hispanic children comprised greater than 50% of the total population of autistic children than in areas in which Hispanic children comprised less than 50% of the total population of autistic children (>50% of the population: β = 0.04; <50% of the population: β = 0.07; *P* < .001). On the other hand, CBSAs in which White children comprised greater than 50% of the total population of autistic children had a significantly greater number of resources per child than CBSAs in which White children comprised less than 50% of the total population of autistic children (>50% of the population: β = 0.07; <50% of the population: β = 0.05; *P* < .001) (Figure 4).

## Discussion

In this cross-sectional study, across all 3 analyses performed (national, metropolitan vs micropolitan CBSAs, and proportion of autistic children in each racial and ethnic group in each CBSA), we found that Black and Hispanic autistic children experienced the highest disparities in access to ASD services. At a national level, Hispanic populations had access to significantly fewer resources per child than White populations. The results of the analysis by proportion of autistic children in each racial and ethnic group was consistent with the national results revealing that CBSAs in which Black or Hispanic autistic children comprised the largest proportion had significantly fewer resources than CBSAs in which Black and Hispanic autistic children did not. Our findings also revealed that CBSAs in which autistic children from minoritized racial and ethnic groups comprised the largest proportion had significantly fewer resources than CBSAs in which White autistic children did, suggesting that minoritized racial and ethnic populations had access to significantly fewer ASD resources overall.

Grouping metropolitan and micropolitan CBSAs revealed that in micropolitan CBSAs, Black and Hispanic autistic children had access to significantly fewer resources than White autistic children. In contrast, the analysis of metropolitan CBSAs revealed that only Hispanic autistic children had access to significantly fewer resources than White autistic children. The metropolitan vs micropolitan analyses suggested that population density had implications for which minoritized racial and ethnic groups experienced disparities in access to ASD services; in more densely populated metropolitan areas across the US, Hispanic children had access to the fewest resources, whereas in more rural areas, both Black and Hispanic children had access to the fewest resources. Error bars on resource estimates for American Indian or Alaska Native and Native Hawaiian or other Pacific Islander children were large, suggesting that resource allocation for these groups may vary substantially across CBSAs. We found that American Indian or Alaska Native autistic children had less access to resources than White autistic children in both the national and metropolitan vs micropolitan analyses; however, this group represented a small proportion (0.7%) of our data set, suggesting that more targeted analyses are necessary to evaluate resource accessibility for this group.

### Future Work

Our study identified areas in the US that had greater disparities in access to ASD diagnostic resources and services. Future work will include higher-level regional and state analyses on the data set because this information could be necessary for legislative reform in autism resource distribution. In addition, although grouping our analysis by CBSAs provided a deeper level of granularity than past work, we did not analyze whether racial disparities existed within each CBSA (eg, between adjacent neighborhoods). A CBSA might have an adequate ratio of children in a particular racial or ethnic category to autism resources, but within the CBSA, the resources may be located far from schools, limiting children from receiving access to care. In this case, the CBSA-level analysis would not accurately reflect disproportionate barriers to accessing care among autistic children from minoritized racial and ethnic groups compared with White autistic children in that CBSA. More granular research is warranted that involves directly computing distances and commuting times between schools and resources to identify intra-CBSA–level disparities. Socioeconomic status could also play an important role in the distribution of resources, which was not accounted for in our analysis by racial and ethnic group.^54,55^ In future studies, it will be important to examine whether areas with populations of higher socioeconomic status have better access to autism resources than areas with populations of lower socioeconomic status.

### Implications of Findings

This study’s findings highlighted the need for further research examining access to ASD resources among racial and ethnic groups with smaller populations in the US. For American Indian or Alaska Native and Native Hawaiian or other Pacific Islander populations, the estimates of resources per child were unreliable, as suggested by the wide error bars (a byproduct of the low counts of these racial and ethnic categories in the CBSAs), which necessitates special attention and further inquiry into the experiences of these underserved populations.

Our results suggested the need for greater resource allocation to Black and Hispanic populations with ASD, particularly in micropolitan areas. One potential solution to address these inequities is through policy reform. Because we highlighted specific CBSAs where significant disparities in access to resources exist, we hope that state and national policy makers, stakeholders in hospital and insurance systems, and local clinicians will use these findings to identify populations in need and increase resources in those areas. However, we acknowledge that geographic barriers to accessing resources are only the first challenge in achieving equitable access to ASD resources. Although an autistic individual may be proximally close to an ASD resource, they may not have insurance coverage or financial resources.^56^ A deeper investigation into specific barriers to care is needed to identify mechanisms by which public health initiatives can better provide individuals with access to diagnostic testing and therapies. Existing government initiatives may also be used to lessen inequities for Black and Hispanic communities.^57^ For example, increased Medicaid coverage for ASD diagnostic testing and therapies nationwide and greater awareness of these types of affordable coverage options could be disseminated to minoritized racial and ethnic groups in CBSAs where disparities exist.

Another solution to bridging these gaps is the use of digital diagnostic testing and therapies. Geographic disparities in access to health care services coupled with the increase in ASD incidence emphasize the potential value of digital health care approaches that work in rural areas and community settings. Progress has been made with the authorization of a digital device for autism diagnosis^58^ and digital therapies for the treatment of attention-deficit/hyperactivity disorder^59^ by the US Food and Drug Administration. Updates to medical policies by commercial insurance companies and state Medicaid programs to cover these digital health care solutions may improve access and equality of care.

### Limitations

This study has several limitations. First, the US Department of Education reported Hispanic or Latino children as a single category, without further distinguishing between Hispanic Black, Hispanic White, non-Hispanic Black, and non-Hispanic White children. This broad categorization makes it difficult to identify the disparities that exist for minoritized racial and ethnic populations such as Hispanic Black children.

In addition, while most children (85.1%-93.6%) in kindergarten through grade 12 with an autism diagnosis were receiving autism services in school,^43^ it is possible that some autistic children were not captured. As a result, our calculations may underestimate the prevalence of autism. Consistent with our hypothesis, the CRDC reports autism prevalence that is lower than that reported by the ADDM, which further illustrates the racial disparities found in our study.^2^ Our use of CBSAs allowed us to identify geographic areas with common access to resources. However, our analysis excluded 5.4% of the population living in rural areas not included in CBSAs. More research on the accessibility of autism resources in those areas is needed.

In terms of autism resource reporting, although GapMap is a comprehensive database, it is not likely to include all autism resources across the US. Some resources in the database may be outdated or closed, and other services may be too new to have been captured. In addition, colocation with autism services does not necessarily mean that those services are being used; other barriers, such as the quality of the service, affordability and insurance coverage, and wait times to access services could impact use, even when services are in close proximity.^42^

## Conclusions

This cross-sectional study mapped and quantified autism resource allocation to minoritized racial and ethnic groups across the US and compared data on autism prevalence from the CDRC data set, broken down by race and ethnicity, with autism health care resource distribution across the US to identify specific CBSAs with significant disparities in access to care. On a national level, Hispanic autistic children experienced the most significant disparities in access to autism services. In comparison with White children, both Black and Hispanic children experienced the greatest disparities in access to ASD resources in micropolitan areas, while Hispanic children experienced the greatest disparities in urban areas. This study’s findings may offer targeted opportunities to address inequities in health care access experienced by minoritized racial and ethnic groups.

## Notes

Supplement 1. **eAppendix 1.** The 6 Racial and Ethnic Categories Defined by the Office of Civil Rights, US Department of Education **eAppendix 2.** Resource Types, Description of Category, and Corresponding Number of Resources in GapMap **eFigure.** Differences in Access to Autism Services by Racial and Ethnic Group **eAppendix 3.** Autism Resources Availability by Racial and Ethnic Group for All Core-Based Statistical Areas in the US

Supplement 2.
Data Sharing Statement
