# Supplementary material for: Racial and Ethnic Disparities in Geographic Access to Autism Resources Across the US
Source: JAMA Netw Open. 2023 Jan 23;6(1):e2251182. doi: 10.1001/jamanetworkopen.2022.51182 (PMC9871799; doi:10.1001/jamanetworkopen.2022.51182)
Supplement: Supplement 1. — eAppendix 1. The 6 Racial and Ethnic Categories Defined by the Office of Civil Rights, US Department of Education eAppendix 2. Resource Types, Description of Category, and Corresponding Number of Resources in GapMap eFigure. Differences in Access to Autism Services by Racial and Ethnic Group eAppendix 3. Autism Resources Availability by Racial and Ethnic Group for All Core-Based Statistical Areas in the US [file jamanetwopen-e2251182-s001.pdf]

Supplemental Online Content

Liu B, Paskov K, Kent J, et al. Racial and ethnic disparities in geographic access to autism resources across the US. *JAMA Netw Open*. 2023;6(1):e2251182. doi:10.1001/jamanetworkopen.2022.51182

eAppendix 1. The 6 Racial and Ethnic Categories Defined by the Office of Civil Rights, US Department of Education

eAppendix 2. Resource Types, Description of Category, and Corresponding Number of Resources in GapMap

eFigure. Differences in Access to Autism Services by Racial and Ethnic Group

eAppendix 3. Autism Resources Availability by Racial and Ethnic Group for All Core-Based Statistical Areas in the US

This supplemental material has been provided by the authors to give readers additional information about their work.

© 2023 Liu B et al. *JAMA Network Open*.

eAppendix 1. The 6 Racial and Ethnic Categories Defined by the Office of Civil Rights, US Department of Education

Racial/Ethnic Category	Definition
American Indian or Alaska Native	A person having origins in any of the original peoples of North and South America (including Central America), and who maintains tribal affiliation or community attachment.
Asian	A person having origins in any of the original peoples of the Far East, Southeast Asia, or the Indian subcontinent including, for example, Cambodia, China, India, Japan, Korea, Malaysia, Pakistan, the Philippine Islands, Thailand, and Vietnam.
Black or African American	A person having origins in any of the black racial groups of Africa. Terms such as "Haitian" or "Negro" can be used in addition to "Black or African American."
Hispanic or Latino	A person of Cuban, Mexican, Puerto Rican, South or Central American, or other Spanish culture or origin, regardless of race. The term, "Spanish origin," can be used in addition to "Hispanic or Latino".
Native Hawaiian or Other Pacific Islander	A person having origins in any of the original peoples of Hawaii, Guam, Samoa, or other Pacific Islands.
White	A person having origins in any of the original peoples of Europe, the middle East, or North Africa.

© 2023 Liu B et al. *JAMA Network Open*.

eAppendix 2. Resource Types, Description of Category, and Corresponding Number of Resources in GapMap

**Resource Type in GapMap**	**Description of Resource**	**Number of Resources**
Diagnostic Service	A center that administers autism diagnoses.	2978
Therapy Service	A center that provides therapy and behavioral type services (ex. ABA) to patients with autism.	10722
Adult Services	Autism services that focus particularly on providing care and assistances to adults.	5128
School Resources	An autism resource that is connected to a school or school system.	15596
Others	An autism resource that does not fall into the prior categories (ex. health, camps, and recreational)	24261

A resource center can be classified under multiple resource types.

© 2023 Liu B et al. *JAMA Network Open*.

eFigure. Differences in Access to Autism Services by Racial and Ethnic Group

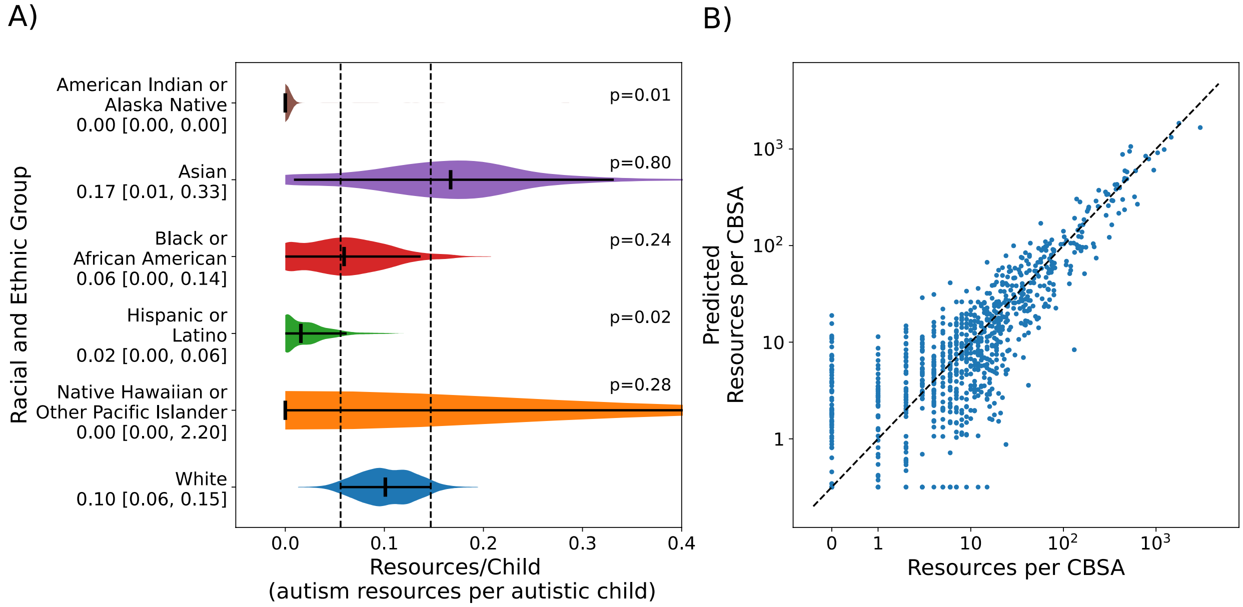

A, shows the bootstrapped distributions of coefficients for each group. Black horizontal lines indicate bootstrapped 95% confidence intervals. Vertical dotted lines show 95% confidence intervals for the majority race and ethnicity group (White). P values stem from comparison of model estimated numbers of resources/child for the race and ethnicity group to the estimated allocations for the White resources/child group. Hispanic or Latino children received significantly fewer resources in comparison to White children. B, shows the fit of the regression model estimates to the raw number of resources per CBSA. *The linear regression with the constraint of nonnegative coefficients makes it such that estimates stemming from race and ethnicity populations who are small in numbers in the United States (eg, American Indian or Alaska Native) could have large confidence intervals and/or resolve to low or even zero estimates in the model. Estimates close to zero indicate that children from this race and ethnicity group have no effect on autism resource allocation.

© 2023 Liu B et al. *JAMA Network Open*.

eAppendix 3. Autism Resources Availability by Racial and Ethnic Group for All Core-Based Statistical Areas in the US

CBSA (searchable address)	CBSA Title (City, State)	Resources per Child (Autism resources/ total autistic children)	American Indian or Alaska Native	Asian	Black or African American	Hispanic or Latino	Native Hawaiian or Other Pacific Islander	White	Total Autistic Children
10700	Albertville, AL Micro Area	0	0	0.007	0.021	0.128	0	0.839	143
10980	Alpena, MI Micro Area	0	0	0	0	0	0	1	28
11380	Andrews, TX Micro Area	0	0	0	0	0.6	0	0.333	15
11580	Arcadia, FL Micro Area	0	0	0	0.135	0.324	0	0.514	37
11660	Arkadelphia, AR Micro Area	0	0	0	0.373	0.034	0	0.477	24
11680	Arkansas City-Winfield, KS Micro Area	0	0.022	0	0.02	0.104	0	0.828	43
12120	Atmore, AL Micro Area	0	0	0	0.295	0	0	0.705	44
12820	Bastrop, LA Micro Area	0	0	0	0.556	0	0	0.444	18
14300	Bonham, TX Micro Area	0	0.024	0	0.064	0.052	0	0.801	33
14380	Boone, NC Micro Area	0	0	0.034	0.017	0.034	0	0.862	58
14420	Borger, TX Micro Area	0	0	0	0.047	0.35	0	0.604	42
14780	Brenham, TX Micro Area	0	0.017	0	0.101	0.356	0	0.525	58
15140	Brownsville, TN Micro Area	0	0	0.033	0.567	0.067	0	0.333	30
15660	Calhoun, GA Micro Area	0	0	0	0.078	0.234	0	0.675	77
15780	Camden, AR Micro Area	0	0	0	0.444	0.008	0	0.526	46
15820	Campbellsville, KY Micro Area	0	0	0	0.026	0	0	0.949	39
15900	Canton, IL Micro Area	0	0.001	0	0	0	0	0.967	88
17060	Chillicothe, OH Micro Area	0	0	0	0.021	0.007	0	0.923	143
17200	Claremont-Lebanon, NH-VT Micro Area	0	0.005	0.005	0.019	0.011	0	0.947	213

© 2023 Liu B et al. *JAMA Network Open*.

17260	Clarksdale, MS Micro Area	0	0	0	0.857	0.029	0	0.114	35
17380	Cleveland, MS Micro Area	0	0	0	0.718	0.026	0.026	0.231	39
17500	Clewiston, FL Micro Area	0	0	0	0.043	0.617	0	0.34	47
17580	Clovis, NM Micro Area	0	0	0.012	0.123	0.414	0	0.402	81
18100	Columbus, NE Micro Area	0	0	0.019	0	0.266	0	0.715	52
18380	Cordele, GA Micro Area	0	0	0	0.346	0.077	0	0.538	26
18780	Craig, CO Micro Area	0	0	0	0	0.176	0	0.706	17
18820	Crawfordsville, IN Micro Area	0	0	0	0	0.094	0	0.875	64
19540	Decatur, IN Micro Area	0	0	0	0.047	0.163	0	0.744	43
19620	Del Rio, TX Micro Area	0	0	0	0	0.855	0	0.132	76
19700	Deming, NM Micro Area	0	0	0	0	0.579	0	0.421	19
19760	DeRidder, LA Micro Area	0	0	0	0.071	0.054	0	0.857	56
20380	Dunn, NC Micro Area	0	0.005	0	0.242	0.167	0	0.532	186
20540	Dyersburg, TN Micro Area	0	0	0	0.167	0.056	0	0.75	36
20580	Eagle Pass, TX Micro Area	0	0.024	0	0	0.969	0	0.008	127
22340	Fitzgerald, GA Micro Area	0	0	0	0.357	0.071	0	0.571	14
22840	Fort Payne, AL Micro Area	0	0.085	0.021	0	0.149	0	0.745	47
23340	Fremont, NE Micro Area	0	0.012	0	0.01	0.135	0.01	0.815	68
24640	Greenfield Town, MA Micro Area	0	0	0.014	0.011	0.074	0	0.832	136
24900	Greenwood, MS Micro Area	0	0	0	0.909	0.03	0	0.061	33
25580	Hastings, NE Micro Area	0	0	0	0.018	0.058	0	0.887	54
25760	Helena-West Helena, AR Micro Area	0	0	0	0.4	0	0	0.6	5
25820	Hereford, TX Micro Area	0	0	0	0	0.571	0	0.381	15
26960	Ionia, MI Micro Area	0	0	0.001	0.009	0.077	0	0.868	94
27660	Jennings, LA Micro Area	0	0	0	0.128	0	0	0.769	39
27920	Junction City, KS Micro Area	0	0.002	0.005	0.218	0.154	0.007	0.546	100
29500	Lamesa, TX Micro Area	0	0.083	0	0	0.513	0	0.405	12
29860	Laurel, MS Micro Area	0	0	0	0.343	0.028	0	0.63	108
29900	Laurinburg, NC Micro Area	0	0.105	0	0.447	0.026	0	0.395	76
29980	Lawrenceburg, TN Micro Area	0	0	0	0	0.049	0	0.951	41

© 2023 Liu B et al. *JAMA Network Open*.

30220	Levelland, TX Micro Area	0	0	0.017	0.067	0.575	0	0.341	63
30820	Lock Haven, PA Micro Area	0	0.001	0	0.047	0.035	0	0.906	80
30880	Logan, WV Micro Area	0	0	0	0	0	0	1	36
31060	Los Alamos, NM Micro Area	0	0.02	0.04	0.02	0.22	0	0.68	49
32020	Marion, OH Micro Area	0	0	0	0.031	0.01	0	0.929	95
32220	Marshall, TX Micro Area	0	0	0.011	0.22	0.172	0	0.552	92
32980	Merrill, WI Micro Area	0	0.011	0	0.011	0.033	0	0.934	90
33420	Mineral Wells, TX Micro Area	0	0.033	0	0.033	0.195	0	0.706	30
34020	Morgan City, LA Micro Area	0	0.014	0	0.304	0.101	0	0.536	69
35060	Natchitoches, LA Micro Area	0	0	0	0.436	0.051	0	0.436	39
36380	Okeechobee, FL Micro Area	0	0	0	0.044	0.267	0	0.611	90
36660	Opelousas, LA Micro Area	0	0.012	0.012	0.512	0	0	0.463	82
36860	Ottawa-Peru, IL Micro Area	0	0	0.015	0.005	0.13	0.003	0.818	200
37020	Owosso, MI Micro Area	0	0.006	0.01	0.001	0.018	0	0.898	100
37080	Oxford, NC Micro Area	0	0	0.025	0.321	0.148	0	0.457	81
37580	Paris, TX Micro Area	0	0	0.012	0.169	0.084	0	0.711	82
38840	Port Clinton, OH Micro Area	0	0	0	0.009	0.061	0	0.888	48
38920	Port Lavaca, TX Micro Area	0	0	0	0	0.682	0	0.318	22
39980	Richmond, IN Micro Area	0	0	0	0.063	0.055	0	0.804	126
40100	Rio Grande City, TX Micro Area	0	0	0	0	0.994	0	0.006	170
40820	Ruston, LA Micro Area	0	0	0.027	0.514	0	0	0.459	37
41820	Sanford, NC Micro Area	0	0.009	0.018	0.155	0.291	0	0.482	110
42820	Selma, AL Micro Area	0	0	0.02	0.857	0	0	0.122	49
43660	Snyder, TX Micro Area	0	0.062	0	0	0.249	0	0.689	16
43940	Spearfish, SD Micro Area	0	0.001	0.001	0	0	0	0.954	23
44920	Summit Park, UT Micro Area	0	0	0	0.022	0.152	0	0.826	46
45740	Toccoa, GA Micro Area	0	0	0	0.026	0.026	0	0.923	39
45980	Troy, AL Micro Area	0	0	0.043	0.362	0.043	0	0.532	47
46740	Valley, AL Micro Area	0	0	0	0.465	0	0	0.535	43
46780	Van Wert, OH Micro Area	0	0.004	0	0	0.05	0	0.93	60
47080	Vidalia, GA Micro Area	0	0	0	0.6	0.1	0	0.3	50
47240	Vineyard Haven, MA Micro Area	0	0.04	0	0.04	0.04	0	0.84	25
47340	Wabash, IN Micro Area	0	0	0.013	0	0.013	0	0.961	77
47920	Washington Court House, OH Micro Area	0	0	0	0.018	0.054	0	0.893	55
49080	Winnemucca, NV Micro Area	0	0.041	0	0.02	0.204	0	0.653	49

© 2023 Liu B et al. *JAMA Network Open*.

25260	Hanford-Corcoran, CA Metro Area	0.0041	0.008	0.036	0.074	0.512	0	0.36	243
15180	Brownsville-Harlingen, TX Metro Area	0.0077	0.001	0.003	0.006	0.955	0	0.032	1041
24420	Grants Pass, OR Metro Area	0.0079	0.008	0	0	0.124	0	0.807	127
30140	Lebanon, PA Metro Area	0.0081	0	0.013	0.03	0.185	0.005	0.753	372
47580	Warner Robins, GA Metro Area	0.0092	0.002	0.007	0.342	0.079	0	0.533	433
38100	Picayune, MS Micro Area	0.01	0	0	0.109	0.007	0	0.867	100
17300	Clarksville, TN-KY Metro Area	0.0104	0.002	0.017	0.228	0.121	0.009	0.557	578
29700	Laredo, TX Metro Area	0.0111	0	0.003	0	0.99	0	0.005	631
31020	Longview, WA Metro Area	0.0111	0	0.006	0	0.092	0.006	0.836	179
49300	Wooster, OH Micro Area	0.0113	0	0.011	0.017	0.011	0	0.909	176
42380	Sayre, PA Micro Area	0.0115	0	0.011	0	0.023	0	0.933	87
34420	Mount Pleasant, TX Micro Area	0.0116	0	0.012	0.174	0.384	0	0.407	86
31260	Lufkin, TX Micro Area	0.0117	0.006	0.018	0.205	0.24	0	0.48	171
23660	Galesburg, IL Micro Area	0.012	0	0.012	0.048	0.078	0	0.805	83
49780	Zanesville, OH Micro Area	0.0128	0	0.005	0	0.025	0	0.918	156
26500	Huntingdon, PA Micro Area	0.0129	0	0	0.009	0	0	0.982	77
34340	Mount Airy, NC Micro Area	0.013	0	0.026	0.013	0.156	0	0.779	77
24980	Grenada, MS Micro Area	0.0132	0	0	0.447	0	0	0.539	76
29020	Kokomo, IN Metro Area	0.0132	0	0.013	0.059	0.046	0	0.803	152
21020	Elizabeth City, NC Micro Area	0.0135	0	0.014	0.378	0.061	0	0.527	148
23860	Georgetown, SC Micro Area	0.0139	0	0	0.583	0	0	0.347	72
39780	Red Bluff, CA Micro Area	0.0141	0.082	0.007	0.021	0.341	0	0.501	141
28780	Kingsville, TX Micro Area	0.0143	0.014	0	0.057	0.743	0	0.186	70
32580	McAllen-Edinburg-Mission, TX Metro Area	0.0146	0.002	0.01	0.002	0.958	0	0.026	1642
40460	Rockingham, NC Micro Area	0.0149	0	0.03	0.269	0.03	0	0.642	67
40580	Rocky Mount, NC Metro Area	0.015	0	0	0.504	0.094	0	0.361	266
18300	Coos Bay, OR Micro Area	0.0152	0.061	0.008	0	0.129	0	0.682	132
13420	Bemidji, MN Micro Area	0.0154	0.257	0.021	0.015	0.026	0	0.594	194
20940	El Centro, CA Metro Area	0.0155	0.016	0.007	0.002	0.914	0	0.052	450
25200	Hailey, ID Micro Area	0.0155	0.001	0	0.003	0.282	0	0.681	64
25900	Hilo, HI Micro Area	0.0155	0.002	0.283	0.019	0.144	0.192	0.205	969
18420	Corinth, MS Micro Area	0.0167	0.017	0	0.267	0.017	0	0.7	60
33300	Milledgeville, GA Micro Area	0.0167	0	0.017	0.7	0	0	0.25	60
41260	St. Marys, PA Micro Area	0.0172	0	0	0.024	0.017	0	0.908	58
15500	Burlington, NC Metro Area	0.0175	0.007	0.011	0.302	0.147	0	0.477	285

© 2023 Liu B et al. *JAMA Network Open*.

18460	Cornelia, GA Micro Area	0.0179	0.018	0.018	0.018	0.125	0	0.804	56
44700	Stockton-Lodi, CA Metro Area	0.0179	0.006	0.142	0.097	0.442	0.004	0.26	1617
10940	Alma, MI Micro Area	0.018	0.001	0	0.018	0.113	0	0.821	55
48980	Wilson, NC Micro Area	0.0183	0.018	0	0.486	0.083	0	0.385	109
24700	Greensburg, IN Micro Area	0.0185	0	0	0	0	0	1	53
37220	Pahrump, NV Micro Area	0.0185	0.037	0.037	0.019	0.185	0	0.704	54
25880	Hillsdale, MI Micro Area	0.0188	0	0	0.004	0.003	0	0.973	53
22100	Farmington, MO Micro Area	0.0189	0	0.009	0	0.028	0	0.944	105
14220	Bogalusa, LA Micro Area	0.0192	0	0	0.365	0.019	0	0.615	52
13300	Beeville, TX Micro Area	0.0198	0	0	0.026	0.784	0	0.191	50
30340	Lewiston-Auburn, ME Metro Area	0.0199	0	0	0.071	0.008	0	0.901	251
37260	Palatka, FL Micro Area	0.0199	0	0.007	0.146	0.166	0	0.629	151
23180	Frankfort, KY Micro Area	0.02	0	0.02	0.13	0.02	0	0.77	100
34260	Mountain Home, AR Micro Area	0.02	0.02	0	0	0.04	0	0.92	50
11220	Amsterdam, NY Micro Area	0.0202	0.018	0.037	0.037	0.295	0	0.517	49
13660	Big Rapids, MI Micro Area	0.0203	0.019	0	0.02	0.013	0	0.912	49
16340	Cedartown, GA Micro Area	0.0208	0	0	0.146	0.167	0	0.646	48
14700	Branson, MO Micro Area	0.0209	0.005	0.011	0.005	0.138	0	0.815	191
42740	Sedalia, MO Micro Area	0.0209	0	0.01	0.063	0.042	0	0.874	95
39740	Reading, PA Metro Area	0.021	0.001	0.026	0.053	0.321	0	0.574	1140
21340	El Paso, TX Metro Area	0.0212	0.003	0.009	0.043	0.819	0.004	0.106	1459
21640	Eufaula, AL-GA Micro Area	0.0213	0	0	0.489	0	0.021	0.489	47
46100	Tullahoma-Manchester, TN Micro Area	0.0213	0.007	0	0.05	0.035	0	0.901	141
38240	Pinehurst-Southern Pines, NC Micro Area	0.0215	0.005	0.016	0.204	0.081	0	0.651	186
31500	Madison, IN Micro Area	0.0217	0	0.022	0.022	0	0	0.913	46
18060	Columbus, MS Micro Area	0.0222	0	0.011	0.567	0	0	0.411	90
33980	Morehead City, NC Micro Area	0.0225	0	0.022	0.011	0.056	0	0.876	89
47620	Warren, PA Micro Area	0.0226	0.011	0.008	0.003	0.008	0	0.937	133
35460	Newport, TN Micro Area	0.0227	0	0	0.023	0.023	0	0.909	44
10860	Alice, TX Micro Area	0.023	0	0	0	0.8	0	0.189	86
32900	Merced, CA Metro Area	0.023	0	0.074	0.034	0.614	0.002	0.244	696
20980	El Dorado, AR Micro Area	0.0231	0	0	0.482	0.105	0	0.39	43
23460	Gadsden, AL Metro Area	0.0231	0.023	0	0.085	0.051	0	0.84	130
22780	Fort Leonard Wood, MO Micro Area	0.0234	0.016	0.031	0.125	0.121	0.008	0.628	128

© 2023 Liu B et al. *JAMA Network Open*.

36780	Oshkosh-Neenah, WI Metro Area	0.0236	0.003	0.022	0.024	0.043	0	0.87	382
19420	Dayton, TN Micro Area	0.0238	0	0.024	0.024	0.024	0	0.929	42
34220	Moultrie, GA Micro Area	0.0238	0	0	0.262	0.214	0	0.5	42
43140	Shelby, NC Micro Area	0.024	0	0	0.293	0.029	0	0.663	208
10540	Albany, OR Metro Area	0.0241	0.018	0.002	0	0.095	0	0.819	332
18580	Corpus Christi, TX Metro Area	0.0242	0.002	0.015	0.028	0.672	0.001	0.261	867
18700	Corvallis, OR Metro Area	0.0242	0.002	0.051	0.006	0.136	0	0.76	165
19140	Dalton, GA Metro Area	0.0242	0	0.008	0.024	0.319	0	0.609	248
47300	Visalia-Porterville, CA Metro Area	0.0244	0.007	0.03	0.014	0.656	0.004	0.274	695
48260	Weirton-Steubenville, WV-OH Metro Area	0.0244	0	0.003	0.017	0.004	0	0.951	287
48460	West Plains, MO Micro Area	0.0244	0.012	0	0	0.024	0	0.927	82
29740	Las Cruces, NM Metro Area	0.0245	0.009	0.006	0.03	0.74	0.003	0.211	326
19980	Dodge City, KS Micro Area	0.0248	0	0.05	0.025	0.495	0	0.396	40
28340	Kendallville, IN Micro Area	0.0248	0	0	0.008	0.07	0	0.872	121
46460	Union City, TN-KY Micro Area	0.025	0	0	0.125	0	0	0.825	40
43900	Spartanburg, SC Metro Area	0.0252	0.004	0.026	0.255	0.063	0.002	0.585	476
32820	Memphis, TN-MS-AR Metro Area	0.0253	0.001	0.022	0.433	0.064	0	0.455	2256
13700	Big Spring, TX Micro Area	0.0255	0	0.051	0.025	0.538	0	0.335	39
46700	Vallejo-Fairfield, CA Metro Area	0.0257	0.001	0.148	0.147	0.296	0.007	0.308	739
20700	East Stroudsburg, PA Metro Area	0.0261	0.008	0.009	0.162	0.216	0.004	0.582	382
33700	Modesto, CA Metro Area	0.0261	0.006	0.063	0.033	0.489	0.004	0.345	1033
36020	Oak Harbor, WA Micro Area	0.0261	0.012	0.026	0.021	0.145	0.01	0.697	191
42620	Searcy, AR Micro Area	0.0261	0	0.009	0.061	0.052	0	0.826	114
42860	Seneca, SC Micro Area	0.0261	0	0.009	0.078	0.078	0	0.809	115
25220	Hammond, LA Metro Area	0.0263	0	0	0.368	0.035	0	0.535	114
44900	Summerville, GA Micro Area	0.0263	0	0	0.026	0.158	0	0.789	38
38380	Plainview, TX Micro Area	0.0264	0	0	0.013	0.747	0	0.239	75
34100	Morristown, TN Metro Area	0.0267	0.007	0.007	0.047	0.093	0	0.84	150
20060	Douglas, GA Micro Area	0.027	0	0	0.351	0.189	0	0.459	37
35440	Newport, OR Micro Area	0.0283	0.026	0	0.017	0.094	0.009	0.718	106
24740	Greenville, MS Micro Area	0.0286	0	0	0.714	0	0	0.286	35
32660	McMinnville, TN Micro Area	0.0286	0	0.029	0.043	0.043	0	0.871	70
26660	Huntsville, TX Micro Area	0.0287	0.01	0.038	0.172	0.182	0	0.56	104
49620	York-Hanover, PA Metro Area	0.0287	0.006	0.017	0.096	0.124	0	0.715	696

© 2023 Liu B et al. *JAMA Network Open*.

34460	Mount Sterling, KY Micro Area	0.029	0.014	0	0.029	0	0	0.942	69
47820	Washington, NC Micro Area	0.029	0	0.014	0.261	0.159	0	0.522	69
32940	Meridian, MS Micro Area	0.0294	0	0	0.493	0.007	0	0.5	136
26420	Houston-The Woodlands-Sugar Land, TX Metro Area	0.0295	0.006	0.08	0.179	0.419	0.001	0.29	14744
12540	Bakersfield, CA Metro Area	0.0298	0.01	0.045	0.067	0.532	0.003	0.325	1812
24140	Goldsboro, NC Metro Area	0.0298	0	0.013	0.391	0.103	0.003	0.444	302
16100	Carlsbad-Artesia, NM Micro Area	0.0299	0.006	0.01	0.036	0.469	0	0.479	100
44500	Stephenville, TX Micro Area	0.0303	0	0	0	0.245	0	0.755	32
45500	Texarkana, TX-AR Metro Area	0.0304	0.008	0.015	0.259	0.057	0.004	0.619	262
28660	Killeen-Temple, TX Metro Area	0.0306	0.01	0.018	0.213	0.269	0.008	0.412	980
22420	Flint, MI Metro Area	0.0307	0.003	0.008	0.16	0.04	0	0.731	715
11140	Americus, GA Micro Area	0.0312	0	0	0.625	0.125	0	0.219	32
29820	Las Vegas-Henderson-Paradise, NV Metro Area	0.0312	0.003	0.071	0.129	0.374	0.009	0.333	4834
21860	Fairmont, MN Micro Area	0.0314	0.016	0	0	0.083	0	0.886	63
43300	Sherman-Denison, TX Metro Area	0.0314	0.022	0.017	0.07	0.13	0	0.697	223
11500	Anniston-Oxford-Jacksonville, AL Metro Area	0.032	0	0	0.218	0.022	0	0.751	218
37420	Pampa, TX Micro Area	0.032	0	0	0.064	0.32	0	0.584	31
14140	Bluefield, WV-VA Micro Area	0.0321	0	0.013	0.083	0.006	0	0.84	156
22220	Fayetteville-Springdale-Rogers, AR-MO Metro Area	0.0321	0.014	0.027	0.025	0.194	0.014	0.69	1369
34180	Moses Lake, WA Micro Area	0.0321	0.017	0.016	0.008	0.459	0	0.467	124
35940	Norwalk, OH Micro Area	0.0321	0	0.022	0	0.082	0	0.864	93
33180	Middlesborough, KY Micro Area	0.0323	0	0	0.065	0.032	0	0.839	31
22620	Forrest City, AR Micro Area	0.0324	0	0.032	0.642	0	0	0.326	30
36700	Orangeburg, SC Micro Area	0.0326	0	0	0.837	0.022	0.011	0.109	92
24620	Greeneville, TN Micro Area	0.0328	0.016	0	0	0	0	0.934	61
40940	Safford, AZ Micro Area	0.0333	0	0	0.017	0.167	0	0.817	60
47260	Virginia Beach-Norfolk-Newport News, VA-NC Metro Area	0.0333	0.002	0.031	0.337	0.09	0.004	0.461	4177
32620	McComb, MS Micro Area	0.0339	0	0	0.559	0	0	0.424	59
14740	Bremerton-Silverdale, WA Metro Area	0.034	0.008	0.039	0.02	0.122	0.006	0.686	646
19100	Dallas-Fort Worth-Arlington, TX Metro Area	0.0341	0.004	0.08	0.171	0.321	0.002	0.39	15638
19660	Deltona-Daytona Beach-Ormond Beach, FL Metro Area	0.0341	0.003	0.017	0.117	0.191	0	0.624	1026
29380	Lake City, FL Micro Area	0.0341	0	0.011	0.17	0.114	0	0.625	88

© 2023 Liu B et al. *JAMA Network Open*.

23420	Fresno, CA Metro Area	0.0343	0.006	0.096	0.05	0.54	0.001	0.263	1691
25060	Gulfport-Biloxi-Pascagoula, MS Metro Area	0.0343	0.004	0.015	0.234	0.052	0.001	0.643	757
17340	Clearlake, CA Micro Area	0.0345	0.052	0	0.017	0.25	0.009	0.603	116
45540	The Villages, FL Metro Area	0.0345	0.017	0	0.155	0.172	0	0.586	58
45180	Talladega-Sylacauga, AL Micro Area	0.0347	0	0	0.327	0.048	0	0.623	86
20900	El Campo, TX Micro Area	0.0349	0.012	0.012	0.055	0.459	0	0.462	86
16420	Central City, KY Micro Area	0.0351	0	0	0.035	0	0	0.895	57
48900	Wilmington, NC Metro Area	0.0351	0.002	0.008	0.211	0.097	0	0.641	513
31340	Lynchburg, VA Metro Area	0.0353	0.005	0.009	0.268	0.033	0.002	0.614	425
12220	Auburn-Opelika, AL Metro Area	0.0354	0.006	0.035	0.266	0.07	0	0.602	254
34820	Myrtle Beach-Conway-North Myrtle Beach, SC-NC Metro Area	0.0356	0.006	0.013	0.135	0.092	0	0.7	533
13140	Beaumont-Port Arthur, TX Metro Area	0.0358	0.008	0.019	0.249	0.14	0	0.558	727
28420	Kennewick-Richland, WA Metro Area	0.0358	0.002	0.011	0.007	0.34	0	0.59	614
27340	Jacksonville, NC Metro Area	0.0359	0.005	0.003	0.205	0.138	0.003	0.549	390
40660	Rome, GA Metro Area	0.0359	0	0.01	0.138	0.067	0	0.754	195
18860	Crescent City, CA Micro Area	0.0364	0.145	0.073	0.018	0.182	0	0.564	55
28620	Kill Devil Hills, NC Micro Area	0.0364	0	0	0.018	0.109	0	0.818	55
36220	Odessa, TX Metro Area	0.0364	0	0.036	0.024	0.618	0	0.309	165
24780	Greenville, NC Metro Area	0.0366	0	0.01	0.439	0.078	0.005	0.431	383
27900	Joplin, MO Metro Area	0.0366	0.021	0.011	0.017	0.078	0	0.827	463
40980	Saginaw, MI Metro Area	0.0366	0.003	0.016	0.233	0.098	0	0.639	300
32500	Maysville, KY Micro Area	0.037	0	0	0	0.074	0	0.815	27
42340	Savannah, GA Metro Area	0.037	0	0.01	0.389	0.071	0	0.466	676
49820	Zapata, TX Micro Area	0.037	0	0	0	0.963	0	0.037	27
49820	Zapata, TX Micro Area	0.037	0.0% (0)	0.0% (0)	0.0% (0)	96.3% (26)	0.0% (0)	3.7% (1)	27
39860	Red Wing, MN Micro Area	0.0372	0.008	0	0.013	0.043	0	0.871	80
41100	St. George, UT Metro Area	0.0373	0.015	0.004	0.011	0.101	0.011	0.836	268
47700	Warsaw, IN Micro Area	0.0374	0.009	0.014	0.016	0.111	0	0.827	213
21700	Eureka-Arcata-Fortuna, CA Micro Area	0.0376	0.088	0.021	0.008	0.121	0	0.666	239
29460	Lakeland-Winter Haven, FL Metro Area	0.0376	0.006	0.014	0.141	0.375	0	0.437	797
38820	Port Angeles, WA Micro Area	0.0376	0.023	0.015	0.038	0.075	0	0.729	133
29540	Lancaster, PA Metro Area	0.0377	0	0.029	0.044	0.166	0	0.725	1035

© 2023 Liu B et al. *JAMA Network Open*.

23980	Glasgow, KY Micro Area	0.038	0	0.013	0.038	0.013	0	0.873	79
49380	Worthington, MN Micro Area	0.0381	0	0.067	0.057	0.238	0	0.629	105
37060	Oxford, MS Micro Area	0.0385	0	0.013	0.308	0.013	0	0.641	78
25980	Hinesville, GA Metro Area	0.0387	0	0.006	0.459	0.122	0.011	0.326	181
17980	Columbus, GA-AL Metro Area	0.0389	0.007	0.006	0.474	0.085	0	0.381	616
13060	Bay City, TX Micro Area	0.0392	0	0	0.123	0.539	0	0.338	50
48180	Waycross, GA Micro Area	0.0392	0.01	0	0.324	0.059	0	0.559	102
24580	Green Bay, WI Metro Area	0.0394	0.03	0.031	0.036	0.115	0.002	0.739	913
28260	Kearney, NE Micro Area	0.0395	0.01	0.02	0	0.146	0	0.823	101
12660	Baraboo, WI Micro Area	0.0396	0	0.019	0	0.088	0	0.881	126
49020	Winchester, VA-WV Metro Area	0.0396	0.006	0.006	0.076	0.119	0.003	0.732	328
46620	Uvalde, TX Micro Area	0.0397	0	0	0	0.794	0	0.206	50
30420	Lexington, NE Micro Area	0.0398	0	0.02	0.013	0.323	0	0.628	50
33620	Moberly, MO Micro Area	0.0398	0	0	0.08	0.02	0	0.861	50
35100	New Bern, NC Metro Area	0.04	0	0.025	0.244	0.116	0	0.556	275
46900	Vernon, TX Micro Area	0.04	0	0	0.04	0.32	0	0.64	25
26300	Hot Springs, AR Metro Area	0.0403	0	0.013	0.082	0.07	0	0.776	148
16380	Celina, OH Micro Area	0.0405	0	0	0	0.02	0.018	0.962	49
27860	Jonesboro, AR Metro Area	0.0406	0	0.016	0.187	0.07	0	0.706	246
19460	Decatur, AL Metro Area	0.0407	0.004	0.016	0.188	0.064	0	0.714	245
22180	Fayetteville, NC Metro Area	0.0407	0.008	0.02	0.388	0.146	0.004	0.359	836
27600	Jefferson, GA Micro Area	0.0407	0	0.049	0.081	0.081	0	0.756	123
21740	Evanston, WY Micro Area	0.0408	0	0	0	0.102	0.02	0.857	49
36940	Owatonna, MN Micro Area	0.0408	0	0.01	0.041	0.067	0	0.861	97
32780	Medford, OR Metro Area	0.0411	0.009	0.004	0.002	0.171	0.002	0.771	534
49180	Winston-Salem, NC Metro Area	0.0414	0.002	0.012	0.239	0.127	0.001	0.576	1039
11020	Altoona, PA Metro Area	0.0417	0.007	0.007	0.034	0.024	0	0.927	288
45380	Taylorville, IL Micro Area	0.0417	0	0.004	0.01	0.022	0	0.928	95
17420	Cleveland, TN Metro Area	0.0419	0	0.024	0.048	0.102	0	0.79	167
40140	Riverside-San Bernardino-Ontario, CA Metro Area	0.0423	0.006	0.072	0.091	0.551	0.003	0.241	8503
39260	Prineville, OR Micro Area	0.0424	0	0	0	0.069	0	0.793	23
11780	Ashtabula, OH Micro Area	0.0425	0.005	0	0.019	0.038	0	0.882	212
14620	Bradford, PA Micro Area	0.0425	0	0	0.037	0	0	0.948	70
15100	Brookings, SD Micro Area	0.0426	0	0	0	0.043	0	0.936	47
32460	Mayfield, KY Micro Area	0.0426	0	0	0.021	0.085	0	0.851	47

© 2023 Liu B et al. *JAMA Network Open*.

46500	Urbana, OH Micro Area	0.0429	0	0	0	0.058	0	0.913	69
48060	Watertown-Fort Drum, NY Metro Area	0.0429	0	0.005	0.043	0.064	0	0.882	186
25860	Hickory-Lenoir-Morganton, NC Metro Area	0.0431	0	0.035	0.078	0.125	0.002	0.72	510
34780	Muskogee, OK Micro Area	0.0433	0.271	0	0.078	0.023	0	0.478	138
34860	Nacogdoches, TX Micro Area	0.0434	0	0	0.233	0.193	0	0.552	92
15020	Brookhaven, MS Micro Area	0.0435	0	0	0.348	0	0	0.652	46
15260	Brunswick, GA Metro Area	0.0435	0.004	0.013	0.3	0.052	0	0.587	230
28820	Kinston, NC Micro Area	0.0435	0	0.011	0.38	0.109	0	0.467	92
23620	Gainesville, TX Micro Area	0.0436	0	0	0.027	0.272	0	0.592	45
24380	Grants, NM Micro Area	0.0437	0.204	0.009	0.044	0.35	0.044	0.306	22
13260	Bedford, IN Micro Area	0.0439	0.009	0	0.009	0.026	0	0.947	114
23940	Gillette, WY Micro Area	0.0439	0.009	0.018	0	0.079	0	0.886	114
44060	Spokane-Spokane Valley, WA Metro Area	0.0439	0.011	0.012	0.019	0.096	0.004	0.782	980
44340	Statesboro, GA Micro Area	0.044	0	0	0.374	0.066	0	0.516	91
49420	Yakima, WA Metro Area	0.0443	0.039	0.005	0.005	0.494	0.003	0.436	384
10300	Adrian, MI Micro Area	0.0451	0	0	0.024	0.109	0	0.838	155
39060	Pottsville, PA Micro Area	0.0451	0.001	0.005	0.02	0.094	0.003	0.848	288
33780	Monroe, MI Metro Area	0.0453	0	0.019	0.008	0.033	0	0.927	154
21460	Enterprise, AL Micro Area	0.0455	0	0.015	0.164	0.074	0	0.717	65
25780	Henderson, NC Micro Area	0.0455	0	0	0.682	0.114	0	0.159	44
46980	Vicksburg, MS Micro Area	0.0455	0	0	0.591	0	0	0.409	66
17780	College Station-Bryan, TX Metro Area	0.0456	0	0.035	0.115	0.322	0	0.494	394
27380	Jacksonville, TX Micro Area	0.0457	0	0.023	0.09	0.298	0	0.571	87
31980	Marion, IN Micro Area	0.0459	0	0.007	0.026	0.063	0	0.847	152
43060	Shawnee, OK Micro Area	0.046	0.157	0	0.041	0.06	0	0.634	130
11420	Angola, IN Micro Area	0.0462	0	0	0.015	0.04	0	0.899	65
22060	Faribault-Northfield, MN Micro Area	0.0463	0.008	0.004	0.051	0.125	0	0.803	151
10820	Alexandria, MN Micro Area	0.0465	0.016	0	0.016	0.062	0	0.876	128
12140	Auburn, IN Micro Area	0.0465	0.023	0	0	0.04	0	0.937	85
27700	Jesup, GA Micro Area	0.0465	0	0	0.209	0.047	0	0.744	43
37300	Palestine, TX Micro Area	0.0465	0	0.031	0.124	0.202	0	0.599	64
23060	Fort Wayne, IN Metro Area	0.0466	0.003	0.019	0.107	0.078	0.001	0.729	750
31740	Manhattan, KS Metro Area	0.0467	0.012	0.023	0.086	0.109	0.002	0.69	149
16180	Carson City, NV Metro Area	0.0469	0.031	0.016	0	0.312	0	0.547	64

© 2023 Liu B et al. *JAMA Network Open*.

19260	Danville, VA Micro Area	0.0469	0	0.014	0.385	0.014	0	0.549	213
22520	Florence-Muscle Shoals, AL Metro Area	0.0469	0	0	0.172	0.047	0	0.724	192
30060	Lebanon, MO Micro Area	0.0469	0	0	0.031	0.019	0	0.93	85
37120	Ozark, AL Micro Area	0.0469	0	0.002	0.318	0.088	0	0.573	63
39460	Punta Gorda, FL Metro Area	0.0469	0.006	0.022	0.072	0.197	0.003	0.653	320
16540	Chambersburg-Waynesboro, PA Metro Area	0.047	0	0.013	0.05	0.068	0	0.814	234
31580	Madisonville, KY Micro Area	0.0476	0.012	0	0.048	0.024	0.012	0.869	84
32280	Martin, TN Micro Area	0.0476	0	0	0.143	0.048	0	0.81	21
41220	St. Marys, GA Micro Area	0.0476	0.012	0	0.155	0.071	0	0.667	84
33460	Minneapolis-St. Paul-Bloomington, MN-WI Metro Area	0.0478	0.008	0.067	0.117	0.078	0.001	0.675	10829
36100	Ocala, FL Metro Area	0.0478	0.004	0.023	0.123	0.227	0.004	0.584	481
17020	Chico, CA Metro Area	0.048	0.014	0.038	0.01	0.226	0	0.671	521
40620	Rolla, MO Micro Area	0.048	0.012	0.048	0.036	0.049	0	0.829	83
24660	Greensboro-High Point, NC Metro Area	0.0481	0.002	0.044	0.383	0.116	0.001	0.412	1371
31460	Madera, CA Metro Area	0.0482	0.016	0.016	0.028	0.584	0	0.307	249
20820	Effingham, IL Micro Area	0.0483	0	0	0	0	0	0.935	41
44860	Sulphur Springs, TX Micro Area	0.0485	0.012	0	0.073	0.121	0	0.758	82
35820	North Platte, NE Micro Area	0.0487	0	0.032	0.022	0.094	0	0.835	61
49700	Yuba City, CA Metro Area	0.0487	0.003	0.074	0.032	0.293	0	0.499	308
48100	Wauchula, FL Micro Area	0.0488	0	0	0.073	0.512	0	0.366	41
18740	Coshocton, OH Micro Area	0.0489	0	0	0.033	0.019	0	0.937	61
27160	Jackson, OH Micro Area	0.0489	0	0.012	0.013	0	0	0.964	81
20180	DuBois, PA Micro Area	0.049	0	0	0.002	0.002	0	0.977	163
19380	Dayton, OH Metro Area	0.0491	0.002	0.022	0.18	0.042	0.002	0.699	1303
48660	Wichita Falls, TX Metro Area	0.0492	0.003	0.023	0.066	0.201	0	0.631	304
40260	Roanoke Rapids, NC Micro Area	0.0494	0.037	0	0.63	0.062	0	0.235	81
37860	Pensacola-Ferry Pass-Brent, FL Metro Area	0.0498	0.005	0.018	0.174	0.071	0.004	0.637	843
23500	Gaffney, SC Micro Area	0.05	0	0.005	0.258	0.041	0	0.669	79
27220	Jackson, WY-ID Micro Area	0.05	0	0	0	0.4	0	0.6	40
44260	Starkville, MS Micro Area	0.05	0	0.033	0.617	0.017	0	0.333	60
12380	Austin, MN Micro Area	0.0501	0.009	0.054	0.054	0.159	0	0.668	99
30620	Lima, OH Metro Area	0.0503	0.003	0	0.125	0.031	0	0.797	159
41420	Salem, OR Metro Area	0.0504	0.016	0.012	0.008	0.267	0.006	0.634	1288
42700	Sebring, FL Metro Area	0.0504	0	0.008	0.109	0.387	0.008	0.462	119

© 2023 Liu B et al. *JAMA Network Open*.

49740	Yuma, AZ Metro Area	0.0507	0.018	0.015	0.021	0.77	0.003	0.17	335
43420	Sierra Vista-Douglas, AZ Metro Area	0.0508	0	0	0.046	0.345	0.005	0.543	197
14100	Bloomsburg-Berwick, PA Metro Area	0.0509	0	0.018	0.018	0.045	0	0.88	98
38500	Plymouth, IN Micro Area	0.0511	0	0.01	0	0.131	0	0.82	97
26780	Hutchinson, MN Micro Area	0.0512	0	0.02	0	0.021	0	0.938	97
35140	Newberry, SC Micro Area	0.0513	0	0	0.282	0.128	0	0.538	39
47220	Vineland-Bridgeton, NJ Metro Area	0.0515	0.009	0.004	0.163	0.403	0	0.352	233
18660	Cortland, NY Micro Area	0.0516	0.006	0	0	0.034	0	0.925	58
31300	Lumberton, NC Micro Area	0.0519	0.23	0	0.319	0.104	0	0.304	135
31700	Manchester-Nashua, NH Metro Area	0.052	0.003	0.035	0.034	0.085	0	0.834	768
34740	Muskegon, MI Metro Area	0.052	0.007	0.01	0.084	0.072	0	0.801	269
27260	Jacksonville, FL Metro Area	0.0523	0.001	0.036	0.253	0.125	0.002	0.535	2522
27500	Janesville-Beloit, WI Metro Area	0.0523	0.004	0.028	0.062	0.159	0	0.712	267
26380	Houma-Thibodaux, LA Metro Area	0.0524	0.057	0.017	0.162	0.048	0	0.655	229
22580	Forest City, NC Micro Area	0.0526	0	0	0.105	0.044	0	0.807	114
17220	Clarksburg, WV Micro Area	0.0531	0	0	0	0.009	0	0.973	113
24860	Greenville-Anderson-Mauldin, SC Metro Area	0.0531	0.002	0.02	0.208	0.103	0.001	0.621	1337
40540	Rock Springs, WY Micro Area	0.0533	0.013	0	0	0.2	0	0.733	75
12260	Augusta-Richmond County, GA-SC Metro Area	0.0534	0.001	0.019	0.406	0.082	0.004	0.422	804
46140	Tulsa, OK Metro Area	0.0534	0.159	0.025	0.074	0.116	0.002	0.537	1442
42980	Seymour, IN Micro Area	0.0538	0	0.022	0	0.14	0	0.753	93
12460	Bainbridge, GA Micro Area	0.0541	0	0	0.514	0.081	0	0.351	37
13340	Bellefontaine, OH Micro Area	0.0543	0	0	0	0	0	0.96	73
26580	Huntington-Ashland, WV-KY-OH Metro Area	0.0544	0.003	0.007	0.025	0.02	0	0.924	606
31080	Los Angeles-Long Beach-Anaheim, CA Metro Area	0.0547	0.002	0.136	0.078	0.541	0.003	0.212	32307
36540	Omaha-Council Bluffs, NE-IA Metro Area	0.0548	0.004	0.024	0.113	0.156	0.001	0.646	1824
22500	Florence, SC Metro Area	0.0549	0.006	0.009	0.595	0.009	0	0.329	328
45060	Syracuse, NY Metro Area	0.0549	0.007	0.025	0.133	0.062	0.002	0.728	1111
43780	South Bend-Mishawaka, IN-MI Metro Area	0.0551	0.009	0.027	0.118	0.093	0.002	0.675	635
18140	Columbus, OH Metro Area	0.0554	0.002	0.039	0.176	0.048	0	0.68	4351
35840	North Port-Sarasota-Bradenton, FL Metro Area	0.0556	0.001	0.025	0.101	0.245	0	0.578	1025

© 2023 Liu B et al. *JAMA Network Open*.

36260	Ogden-Clearfield, UT Metro Area	0.0557	0.007	0.006	0.016	0.112	0.003	0.824	1148
41060	St. Cloud, MN Metro Area	0.0557	0.004	0.014	0.054	0.036	0.002	0.851	520
43100	Sheboygan, WI Metro Area	0.0558	0.003	0.078	0.029	0.104	0	0.754	214
41500	Salinas, CA Metro Area	0.0559	0.003	0.072	0.025	0.614	0.002	0.239	590
44620	Stevens Point, WI Micro Area	0.0559	0.007	0.037	0.011	0.065	0	0.864	179
15740	Cambridge, OH Micro Area	0.056	0	0	0	0.006	0	0.976	71
35900	North Wilkesboro, NC Micro Area	0.0563	0	0.014	0.056	0.056	0	0.803	71
39820	Redding, CA Metro Area	0.0563	0.084	0.017	0.017	0.113	0.013	0.689	231
43180	Shelbyville, TN Micro Area	0.0563	0	0	0.113	0.113	0	0.718	71
40780	Russellville, AR Micro Area	0.0566	0.005	0	0.015	0.139	0	0.83	194
26140	Homosassa Springs, FL Metro Area	0.0567	0.007	0.014	0.043	0.149	0	0.73	141
13380	Bellingham, WA Metro Area	0.0568	0.007	0.025	0.019	0.143	0	0.72	317
10180	Abilene, TX Metro Area	0.0571	0	0.015	0.057	0.282	0	0.608	262
21980	Fallon, NV Micro Area	0.0571	0.086	0	0	0.171	0	0.657	35
26540	Huntington, IN Micro Area	0.0571	0	0	0	0.043	0	0.943	70
47540	Wapakoneta, OH Micro Area	0.0572	0.004	0	0.001	0.015	0	0.965	69
26020	Hobbs, NM Micro Area	0.0575	0.011	0	0.046	0.494	0	0.448	87
41400	Salem, OH Micro Area	0.0575	0.001	0	0.009	0.016	0	0.949	191
35860	North Vernon, IN Micro Area	0.0577	0	0	0	0.019	0	0.904	52
21140	Elkhart-Goshen, IN Metro Area	0.0581	0.003	0.025	0.058	0.247	0	0.616	361
24260	Grand Island, NE Metro Area	0.0581	0.013	0.006	0.032	0.175	0	0.741	154
36980	Owensboro, KY Metro Area	0.0581	0	0.017	0.052	0.052	0	0.814	172
30980	Longview, TX Metro Area	0.0582	0.009	0.006	0.162	0.182	0	0.598	343
46660	Valdosta, GA Metro Area	0.0584	0	0.004	0.385	0.054	0.004	0.502	257
37460	Panama City, FL Metro Area	0.0585	0.003	0.023	0.117	0.085	0.003	0.699	342
20300	Dumas, TX Micro Area	0.0587	0.059	0	0	0.587	0	0.354	34
35420	New Philadelphia-Dover, OH Micro Area	0.0587	0	0	0	0.021	0	0.979	119
25180	Hagerstown-Martinsburg, MD-WV Metro Area	0.0591	0	0.011	0.118	0.057	0	0.749	474
31420	Macon-Bibb County, GA Metro Area	0.0591	0	0.005	0.558	0.036	0	0.362	389
18980	Cullman, AL Micro Area	0.0592	0.01	0	0	0.069	0	0.881	101
25620	Hattiesburg, MS Metro Area	0.0592	0	0.007	0.43	0.026	0	0.522	270
31820	Manitowoc, WI Micro Area	0.0596	0.006	0.044	0.034	0.086	0	0.787	167
16860	Chattanooga, TN-GA Metro Area	0.0598	0.003	0.014	0.136	0.047	0.005	0.775	1053
15420	Burley, ID Micro Area	0.06	0.028	0	0	0.237	0	0.722	66

© 2023 Liu B et al. *JAMA Network Open*.

10460	Alamogordo, NM Micro Area	0.0602	0.07	0.01	0.011	0.255	0	0.594	99
34580	Mount Vernon-Anacortes, WA Metro Area	0.0602	0.016	0.027	0.011	0.191	0.016	0.696	182
14540	Bowling Green, KY Metro Area	0.0603	0.003	0.025	0.063	0.098	0	0.787	315
22900	Fort Smith, AR-OK Metro Area	0.0603	0.163	0.037	0.032	0.116	0	0.563	431
40080	Richmond-Berea, KY Micro Area	0.0604	0	0.013	0.047	0.034	0	0.866	149
45300	Tampa-St. Petersburg-Clearwater, FL Metro Area	0.0605	0.002	0.03	0.147	0.281	0.001	0.496	5021
17900	Columbia, SC Metro Area	0.0606	0.002	0.022	0.431	0.059	0.001	0.439	1683
42200	Santa Maria-Santa Barbara, CA Metro Area	0.0606	0.008	0.039	0.012	0.565	0.003	0.326	726
44940	Sumter, SC Metro Area	0.0606	0	0	0.523	0.008	0	0.432	132
45620	Thomasville, GA Micro Area	0.0606	0	0	0.515	0.04	0	0.354	99
38860	Portland-South Portland, ME Metro Area	0.0608	0.006	0.02	0.035	0.027	0.001	0.878	1234
10660	Albert Lea, MN Micro Area	0.061	0.006	0.031	0.018	0.125	0	0.789	180
29060	Laconia, NH Micro Area	0.0611	0	0.01	0.011	0.014	0.004	0.961	98
28860	Kirksville, MO Micro Area	0.0612	0	0	0	0	0	1	49
40900	Sacramento--Roseville--Arden-Arcade, CA Metro Area	0.0615	0.006	0.15	0.092	0.236	0.008	0.432	4637
39580	Raleigh, NC Metro Area	0.0616	0.003	0.069	0.252	0.119	0.001	0.512	3020
40060	Richmond, VA Metro Area	0.0616	0.003	0.041	0.335	0.081	0.001	0.492	2939
38060	Phoenix-Mesa-Scottsdale, AZ Metro Area	0.0618	0.026	0.034	0.066	0.351	0.003	0.485	7324
46220	Tuscaloosa, AL Metro Area	0.062	0.003	0.003	0.491	0.034	0.003	0.46	387
37340	Palm Bay-Melbourne-Titusville, FL Metro Area	0.0621	0	0.021	0.096	0.139	0.001	0.665	998
42540	Scranton--Wilkes-Barre--Hazleton, PA Metro Area	0.0622	0.002	0.005	0.061	0.167	0	0.731	852
39300	Providence-Warwick, RI-MA Metro Area	0.0623	0.005	0.022	0.055	0.138	0	0.737	3018
23580	Gainesville, GA Metro Area	0.0624	0.007	0.016	0.116	0.266	0	0.578	304
18620	Corsicana, TX Micro Area	0.0632	0.011	0.012	0.14	0.269	0	0.516	94
15220	Brownwood, TX Micro Area	0.0633	0	0.032	0.047	0.301	0	0.589	63
19300	Daphne-Fairhope-Foley, AL Metro Area	0.0633	0	0.01	0.077	0.073	0	0.79	300
21300	Elmira, NY Metro Area	0.0634	0.019	0.009	0.08	0.068	0	0.784	126
42460	Scottsboro, AL Micro Area	0.0635	0.063	0	0.048	0.016	0	0.857	63
20260	Duluth, MN-WI Metro Area	0.0638	0.04	0.006	0.019	0.019	0	0.833	627
42780	Selinsgrove, PA Micro Area	0.0638	0	0.021	0	0.043	0	0.915	47
44420	Staunton-Waynesboro, VA Metro Area	0.0638	0.007	0.014	0.035	0.043	0	0.809	141

© 2023 Liu B et al. *JAMA Network Open*.

21660	Eugene, OR Metro Area	0.064	0.007	0.017	0.017	0.121	0.001	0.752	781
41740	San Diego-Carlsbad, CA Metro Area	0.064	0.006	0.124	0.056	0.399	0.004	0.344	7565
30900	Logansport, IN Micro Area	0.0641	0	0.038	0	0.231	0	0.692	78
44300	State College, PA Metro Area	0.0646	0.009	0.043	0.032	0.038	0.009	0.848	232
10900	Allentown-Bethlehem-Easton, PA-NJ Metro Area	0.0653	0.002	0.022	0.088	0.295	0.002	0.555	1654
16700	Charleston-North Charleston, SC Metro Area	0.0653	0.002	0.017	0.336	0.067	0	0.517	1210
42660	Seattle-Tacoma-Bellevue, WA Metro Area	0.0653	0.006	0.127	0.073	0.13	0.006	0.563	7063
43020	Shawano, WI Micro Area	0.0653	0.219	0.009	0	0.052	0	0.693	137
13220	Beckley, WV Metro Area	0.0656	0	0.016	0.082	0.008	0	0.861	122
15340	Bucyrus, OH Micro Area	0.0659	0	0.016	0	0	0.033	0.951	60
25300	Hannibal, MO Micro Area	0.0659	0	0	0.057	0.019	0	0.843	106
21820	Fairbanks, AK Metro Area	0.0662	0.106	0.013	0.026	0.06	0	0.642	151
23140	Frankfort, IN Micro Area	0.0664	0.017	0.012	0	0.149	0	0.805	60
14660	Brainerd, MN Micro Area	0.0665	0.05	0.005	0.015	0.01	0	0.873	405
31900	Mansfield, OH Metro Area	0.0667	0	0.005	0.074	0.01	0	0.85	194
36740	Orlando-Kissimmee-Sanford, FL Metro Area	0.0668	0.002	0.046	0.159	0.41	0.001	0.354	4984
12060	Atlanta-Sandy Springs-Roswell, GA Metro Area	0.0671	0.002	0.063	0.406	0.128	0.001	0.364	11527
28180	Kapaa, HI Micro Area	0.0671	0.002	0.283	0.019	0.144	0.192	0.205	149
26860	Indiana, PA Micro Area	0.0672	0	0	0.025	0.003	0	0.952	163
23700	Gallup, NM Micro Area	0.0673	0.677	0.043	0.011	0.157	0	0.101	89
43760	Sonora, CA Micro Area	0.0676	0.027	0.041	0	0.095	0	0.757	74
32180	Marshall, MO Micro Area	0.0683	0.034	0	0.068	0.102	0	0.795	29
15680	California-Lexington Park, MD Metro Area	0.0686	0	0.01	0.176	0.088	0	0.627	204
30020	Lawton, OK Metro Area	0.0686	0.056	0.007	0.151	0.161	0.003	0.546	291
17140	Cincinnati, OH-KY-IN Metro Area	0.0687	0.002	0.027	0.135	0.044	0.001	0.75	3276
26220	Hood River, OR Micro Area	0.069	0	0	0.069	0.448	0	0.414	29
32300	Martinsville, VA Micro Area	0.069	0.011	0.011	0.299	0.069	0	0.552	87
38180	Pierre, SD Micro Area	0.069	0.172	0	0	0.069	0	0.759	29
10620	Albemarle, NC Micro Area	0.0694	0	0.014	0.139	0.111	0	0.667	72
20140	Dublin, GA Micro Area	0.0694	0	0.014	0.514	0.028	0	0.417	72
39340	Provo-Orem, UT Metro Area	0.0694	0.001	0.009	0.013	0.141	0.006	0.79	821
36420	Oklahoma City, OK Metro Area	0.0695	0.053	0.032	0.101	0.151	0	0.567	2343
29260	La Grande, OR Micro Area	0.0697	0	0	0.023	0.012	0.012	0.872	86

© 2023 Liu B et al. *JAMA Network Open*.

11900	Athens, OH Micro Area	0.0698	0	0.04	0.02	0.04	0	0.855	100
33060	Miami, OK Micro Area	0.0704	0.31	0	0.028	0.042	0	0.535	71
24820	Greenville, OH Micro Area	0.0707	0	0.014	0.001	0	0.002	0.968	70
42680	Sebastian-Vero Beach, FL Metro Area	0.0711	0.005	0.02	0.112	0.162	0.005	0.675	197
19220	Danville, KY Micro Area	0.0714	0	0.012	0.012	0.024	0	0.905	84
15980	Cape Coral-Fort Myers, FL Metro Area	0.0717	0.003	0.024	0.121	0.31	0.001	0.501	1046
21060	Elizabethtown-Fort Knox, KY Metro Area	0.0717	0.004	0.004	0.117	0.06	0.004	0.747	265
40700	Roseburg, OR Micro Area	0.0717	0.022	0.008	0.007	0.079	0	0.855	139
11940	Athens, TN Micro Area	0.0723	0	0	0.048	0.06	0	0.867	83
16820	Charlottesville, VA Metro Area	0.0725	0.003	0.022	0.185	0.075	0	0.66	400
20020	Dothan, AL Metro Area	0.0726	0	0.005	0.317	0.047	0	0.592	179
48620	Wichita, KS Metro Area	0.0726	0.008	0.039	0.094	0.142	0	0.651	1019
18260	Cookeville, TN Micro Area	0.073	0	0	0.022	0.056	0	0.899	178
24940	Greenwood, SC Micro Area	0.073	0	0.031	0.349	0.073	0	0.547	95
49340	Worcester, MA-CT Metro Area	0.0734	0.001	0.051	0.044	0.184	0	0.677	2274
26900	Indianapolis-Carmel-Anderson, IN Metro Area	0.074	0.001	0.024	0.15	0.083	0	0.694	5119
21260	Ellensburg, WA Micro Area	0.0742	0	0	0.037	0.06	0.019	0.864	53
39020	Portsmouth, OH Micro Area	0.0745	0.007	0.007	0.027	0.007	0	0.932	147
12980	Battle Creek, MI Metro Area	0.0748	0.015	0	0.067	0.041	0	0.792	120
10760	Alexander City, AL Micro Area	0.075	0.019	0	0.432	0.037	0	0.512	53
19820	Detroit-Warren-Dearborn, MI Metro Area	0.075	0.003	0.048	0.167	0.045	0.001	0.703	6483
22820	Fort Morgan, CO Micro Area	0.0752	0.038	0	0	0.451	0	0.511	26
18880	Crestview-Fort Walton Beach-Destin, FL Metro Area	0.0756	0.004	0.011	0.109	0.055	0.002	0.731	476
26620	Huntsville, AL Metro Area	0.0758	0.013	0.022	0.249	0.065	0.002	0.603	936
31180	Lubbock, TX Metro Area	0.0759	0.002	0.023	0.097	0.411	0	0.44	474
44780	Sturgis, MI Micro Area	0.0759	0	0.019	0.019	0.099	0	0.754	52
31930	Marietta, OH Micro Area	0.076	0.008	0.008	0	0.017	0	0.927	118
24340	Grand Rapids-Wyoming, MI Metro Area	0.0765	0.002	0.03	0.093	0.098	0.001	0.727	1228
33100	Miami-Fort Lauderdale-West Palm Beach, FL Metro Area	0.0766	0.003	0.026	0.219	0.491	0.001	0.239	12339
18220	Connersville, IN Micro Area	0.0769	0	0	0	0.038	0	0.962	26
43320	Show Low, AZ Micro Area	0.0771	0.258	0	0.039	0.096	0	0.559	103
39380	Pueblo, CO Metro Area	0.0772	0.003	0.003	0.026	0.466	0	0.482	311

© 2023 Liu B et al. *JAMA Network Open*.

13980	Blacksburg-Christiansburg-Radford, VA Metro Area	0.0773	0.005	0.009	0.05	0.032	0	0.882	220
34980	Nashville-Davidson--Murfreesboro--Franklin, TN Metro Area	0.0775	0	0.029	0.197	0.107	0.001	0.649	3057
22260	Fergus Falls, MN Micro Area	0.078	0.059	0.004	0.015	0.041	0	0.796	269
37620	Parkersburg-Vienna, WV Metro Area	0.0783	0	0.009	0.017	0.017	0	0.913	115
16980	Chicago-Naperville-Elgin, IL-IN-WI Metro Area	0.0784	0.002	0.068	0.179	0.268	0.002	0.447	18608
22540	Fond du Lac, WI Metro Area	0.0785	0.003	0.021	0.016	0.054	0.005	0.883	216
49660	Youngstown-Warren-Boardman, OH-PA Metro Area	0.0786	0.001	0.004	0.12	0.039	0	0.792	1005
38740	Poplar Bluff, MO Micro Area	0.0787	0	0	0.034	0	0	0.933	89
21500	Erie, PA Metro Area	0.0791	0.002	0.015	0.072	0.048	0.003	0.831	619
38580	Point Pleasant, WV-OH Micro Area	0.0791	0	0	0.026	0.009	0	0.93	113
10740	Albuquerque, NM Metro Area	0.0792	0.041	0.018	0.019	0.511	0.004	0.356	1351
34900	Napa, CA Metro Area	0.0793	0	0.152	0.043	0.387	0.001	0.318	239
48700	Williamsport, PA Metro Area	0.0794	0.01	0.02	0.034	0.02	0	0.882	201
26090	Holland, MI Micro Area	0.0796	0	0.005	0.013	0.128	0.001	0.842	138
31140	Louisville/Jefferson County, KY-IN Metro Area	0.0796	0.001	0.018	0.173	0.059	0	0.707	1896
41180	St. Louis, MO-IL Metro Area	0.0796	0.003	0.028	0.212	0.038	0.001	0.683	5756
25420	Harrisburg-Carlisle, PA Metro Area	0.0797	0.001	0.039	0.118	0.104	0.001	0.676	1267
33220	Midland, MI Metro Area	0.0798	0	0.03	0.01	0.057	0	0.869	100
24540	Greeley, CO Metro Area	0.0799	0.007	0.024	0.008	0.298	0.001	0.633	375
38900	Portland-Vancouver-Hillsboro, OR-WA Metro Area	0.0799	0.007	0.074	0.033	0.151	0.005	0.657	4631
16220	Casper, WY Metro Area	0.08	0.008	0.008	0.016	0.056	0	0.88	125
39700	Raymondville, TX Micro Area	0.0801	0	0	0	0.965	0	0.035	24
39140	Prescott, AZ Metro Area	0.0804	0.024	0.009	0.024	0.189	0.005	0.717	211
28700	Kingsport-Bristol-Bristol, TN-VA Metro Area	0.0805	0.009	0.002	0.025	0.025	0	0.917	447
24020	Glens Falls, NY Metro Area	0.0812	0.001	0.005	0.016	0.026	0	0.917	197
41700	San Antonio-New Braunfels, TX Metro Area	0.0814	0.002	0.024	0.077	0.595	0.003	0.267	4862
22140	Farmington, NM Metro Area	0.0815	0.393	0	0.022	0.193	0	0.363	135
13820	Birmingham-Hoover, AL Metro Area	0.0819	0.002	0.011	0.287	0.048	0.001	0.637	1745
35700	Nogales, AZ Micro Area	0.082	0	0.016	0	0.918	0	0.066	61
15620	Cadillac, MI Micro Area	0.0829	0	0.001	0	0	0	0.957	48

© 2023 Liu B et al. *JAMA Network Open*.

29420	Lake Havasu City-Kingman, AZ Metro Area	0.0831	0.024	0.004	0.016	0.226	0.004	0.709	252
11540	Appleton, WI Metro Area	0.0837	0.021	0.04	0.013	0.07	0	0.828	609
40740	Roswell, NM Micro Area	0.0838	0.006	0.056	0.02	0.543	0	0.375	71
36500	Olympia-Tumwater, WA Metro Area	0.084	0.009	0.04	0.042	0.142	0.01	0.655	559
46300	Twin Falls, ID Metro Area	0.084	0.007	0	0.034	0.175	0	0.77	142
41940	San Jose-Sunnyvale-Santa Clara, CA Metro Area	0.0844	0.005	0.419	0.026	0.264	0.002	0.221	3470
38220	Pine Bluff, AR Metro Area	0.0845	0	0	0.557	0.05	0	0.368	82
11100	Amarillo, TX Metro Area	0.0847	0.003	0.034	0.041	0.271	0	0.603	295
34540	Mount Vernon, OH Micro Area	0.0849	0	0.011	0.04	0.042	0	0.865	94
27980	Kahului-Wailuku-Lahaina, HI Metro Area	0.085	0.002	0.283	0.019	0.144	0.192	0.205	282
17660	Coeur d'Alene, ID Metro Area	0.0851	0.008	0.008	0.012	0.081	0	0.854	246
28380	Kennett, MO Micro Area	0.0851	0	0	0.043	0.043	0	0.894	47
40220	Roanoke, VA Metro Area	0.0854	0	0.016	0.202	0.054	0	0.701	609
11980	Athens, TX Micro Area	0.0856	0.026	0	0.067	0.146	0	0.734	81
41140	St. Joseph, MO-KS Metro Area	0.0856	0	0	0.051	0.029	0	0.883	105
27540	Jasper, IN Micro Area	0.0857	0.01	0.01	0.01	0.067	0	0.857	105
49220	Wisconsin Rapids-Marshfield, WI Micro Area	0.0858	0.008	0.053	0	0.037	0	0.87	93
33260	Midland, TX Metro Area	0.0859	0	0.019	0.072	0.533	0	0.367	209
41860	San Francisco-Oakland-Hayward, CA Metro Area	0.0859	0.003	0.292	0.101	0.255	0.006	0.261	7007
33660	Mobile, AL Metro Area	0.0865	0.007	0.01	0.349	0.046	0.002	0.555	416
41540	Salisbury, MD-DE Metro Area	0.0866	0	0.014	0.197	0.108	0	0.638	623
31860	Mankato-North Mankato, MN Metro Area	0.0867	0	0.01	0.039	0.058	0	0.864	288
30300	Lewiston, ID-WA Metro Area	0.0872	0.096	0.009	0.009	0.08	0	0.755	103
43340	Shreveport-Bossier City, LA Metro Area	0.0873	0.002	0.016	0.44	0.036	0.005	0.484	550
18020	Columbus, IN Metro Area	0.0876	0	0.102	0.029	0.088	0	0.752	137
44220	Springfield, OH Metro Area	0.0876	0	0	0.088	0.058	0	0.806	205
41660	San Angelo, TX Metro Area	0.0878	0.007	0.014	0.054	0.399	0	0.514	148
15940	Canton-Massillon, OH Metro Area	0.088	0.004	0.008	0.064	0.024	0	0.848	659
48780	Williston, ND Micro Area	0.0882	0.029	0.059	0.059	0.044	0	0.794	68
16500	Centralia, WA Micro Area	0.0883	0.025	0.007	0.013	0.093	0.001	0.836	147
16260	Cedar City, UT Micro Area	0.0886	0.025	0	0	0.114	0	0.848	79
21580	EspaÃ±ola, NM Micro Area	0.0886	0.118	0.028	0.028	0.717	0	0.109	33
22020	Fargo, ND-MN Metro Area	0.0886	0.03	0.015	0.039	0.047	0.004	0.817	541

© 2023 Liu B et al. *JAMA Network Open*.

16740	Charlotte-Concord-Gastonia, NC-SC Metro Area	0.0889	0.004	0.04	0.299	0.137	0.002	0.486	3444
46340	Tyler, TX Metro Area	0.0889	0.009	0.028	0.234	0.178	0	0.529	326
40380	Rochester, NY Metro Area	0.0893	0.001	0.027	0.178	0.142	0.002	0.625	1343
12620	Bangor, ME Metro Area	0.0897	0.021	0.003	0.018	0.018	0.003	0.905	390
40420	Rockford, IL Metro Area	0.09	0.004	0.02	0.114	0.178	0	0.625	466
25500	Harrisonburg, VA Metro Area	0.0902	0	0.008	0.068	0.173	0	0.654	133
35660	Niles-Benton Harbor, MI Metro Area	0.0903	0.015	0.011	0.132	0.046	0	0.751	99
25460	Harrison, AR Micro Area	0.0908	0	0	0.023	0.045	0	0.932	44
37780	Pecos, TX Micro Area	0.0909	0	0	0	0.909	0	0.091	11
43460	Sikeston, MO Micro Area	0.0909	0	0	0.114	0	0	0.795	44
45460	Terre Haute, IN Metro Area	0.0909	0	0.003	0.029	0.024	0	0.89	374
34380	Mount Pleasant, MI Micro Area	0.091	0.045	0.03	0.015	0.034	0	0.832	131
20740	Eau Claire, WI Metro Area	0.0911	0	0.023	0.03	0.041	0.003	0.868	329
32740	Meadville, PA Micro Area	0.0911	0.001	0.005	0.017	0.015	0.005	0.925	175
14460	Boston-Cambridge-Newton, MA-NH Metro Area	0.0913	0.001	0.08	0.098	0.142	0.001	0.645	9132
37980	Philadelphia-Camden-Wilmington, PA-NJ-DE-MD Metro Area	0.0913	0.001	0.062	0.215	0.096	0.001	0.552	11389
13020	Bay City, MI Metro Area	0.0916	0	0.013	0	0.038	0	0.897	76
41620	Salt Lake City, UT Metro Area	0.0918	0.009	0.018	0.022	0.171	0.017	0.732	1732
44580	Sterling, IL Micro Area	0.0919	0	0	0	0.184	0	0.739	87
38300	Pittsburgh, PA Metro Area	0.0924	0.003	0.025	0.132	0.018	0.001	0.78	4371
40340	Rochester, MN Metro Area	0.0927	0.014	0.08	0.077	0.05	0	0.744	399
45000	Susanville, CA Micro Area	0.0929	0.046	0	0.07	0.151	0	0.688	43
21780	Evansville, IN-KY Metro Area	0.0932	0	0.015	0.059	0.034	0	0.841	472
26820	Idaho Falls, ID Metro Area	0.0932	0.013	0.008	0.012	0.1	0	0.818	235
30940	London, KY Micro Area	0.0933	0	0	0.007	0.02	0	0.967	150
31660	Malone, NY Micro Area	0.0934	0.073	0	0	0	0	0.927	64
21900	Fairmont, WV Micro Area	0.0938	0	0	0.062	0	0	0.875	64
39900	Reno, NV Metro Area	0.0941	0.016	0.046	0.025	0.26	0.009	0.567	691
47900	Washington-Arlington-Alexandria, DC-VA-MD-WV Metro Area	0.0947	0.002	0.111	0.256	0.181	0.002	0.395	12938
47020	Victoria, TX Metro Area	0.095	0	0.019	0.054	0.58	0	0.338	105
14010	Bloomington, IL Metro Area	0.0951	0.013	0.067	0.105	0.065	0.003	0.672	388
28140	Kansas City, MO-KS Metro Area	0.0952	0.003	0.03	0.15	0.116	0.002	0.65	3603
38940	Port St. Lucie, FL Metro Area	0.0956	0.004	0.019	0.16	0.245	0	0.536	774

© 2023 Liu B et al. *JAMA Network Open*.

45660	Tiffin, OH Micro Area	0.0961	0	0	0	0.074	0	0.906	104
27740	Johnson City, TN Metro Area	0.0969	0	0.01	0.051	0.02	0	0.913	196
46380	Ukiah, CA Micro Area	0.0969	0.075	0.015	0.015	0.281	0	0.548	134
20340	Duncan, OK Micro Area	0.0971	0.144	0.019	0.057	0.055	0	0.702	51
29200	Lafayette-West Lafayette, IN Metro Area	0.0973	0.003	0.036	0.027	0.103	0	0.799	369
47380	Waco, TX Metro Area	0.0973	0	0.026	0.19	0.287	0	0.471	318
27180	Jackson, TN Metro Area	0.0977	0	0.023	0.368	0.057	0	0.546	174
44180	Springfield, MO Metro Area	0.0981	0.006	0.01	0.023	0.035	0	0.877	672
25840	Hermiston-Pendleton, OR Micro Area	0.0985	0.049	0	0.005	0.379	0	0.527	202
48540	Wheeling, WV-OH Metro Area	0.0985	0	0.016	0.024	0	0	0.953	253
35220	New Castle, IN Micro Area	0.0987	0	0	0.033	0.022	0	0.921	91
10780	Alexandria, LA Metro Area	0.0988	0	0.031	0.358	0.037	0	0.562	162
44980	Sunbury, PA Micro Area	0.099	0	0.008	0.01	0.104	0	0.832	151
38260	Pittsburg, KS Micro Area	0.0993	0.002	0	0	0.099	0.017	0.833	60
37100	Oxnard-Thousand Oaks-Ventura, CA Metro Area	0.0994	0.004	0.081	0.019	0.484	0.001	0.379	1619
12680	Bardstown, KY Micro Area	0.1	0	0.014	0.043	0.029	0	0.914	70
42420	Scottsbluff, NE Micro Area	0.1	0.033	0.033	0	0.167	0	0.767	60
44140	Springfield, MA Metro Area	0.1	0.002	0.019	0.08	0.34	0	0.531	1490
45700	Tifton, GA Micro Area	0.1	0	0	0.3	0.125	0	0.55	40
27140	Jackson, MS Metro Area	0.1003	0	0.017	0.511	0.019	0.001	0.437	698
21120	Elk City, OK Micro Area	0.1004	0.034	0	0.067	0.266	0	0.632	29
29940	Lawrence, KS Metro Area	0.1006	0.037	0.021	0.047	0.088	0	0.728	178
11460	Ann Arbor, MI Metro Area	0.1007	0.002	0.07	0.167	0.06	0.001	0.633	744
31540	Madison, WI Metro Area	0.1009	0.004	0.062	0.06	0.111	0	0.709	1446
48140	Wausau, WI Metro Area	0.1009	0.007	0.068	0.014	0.062	0	0.818	287
27100	Jackson, MI Metro Area	0.1011	0	0.014	0.078	0.04	0	0.83	207
30460	Lexington-Fayette, KY Metro Area	0.1014	0.003	0.024	0.158	0.07	0	0.707	789
33140	Michigan City-La Porte, IN Metro Area	0.1016	0	0.005	0.067	0.088	0	0.797	196
39660	Rapid City, SD Metro Area	0.1024	0.131	0.016	0.004	0.074	0.004	0.718	244
38460	Plattsburgh, NY Micro Area	0.1028	0	0	0.008	0.04	0	0.945	126
33020	Mexico, MO Micro Area	0.1029	0	0	0.026	0.026	0	0.863	38
26340	Houghton, MI Micro Area	0.1034	0.034	0	0	0.034	0	0.897	29
45520	The Dalles, OR Micro Area	0.1034	0.155	0	0	0.241	0.017	0.552	58
45580	Thomaston, GA Micro Area	0.1034	0	0.034	0.448	0	0	0.414	29

© 2023 Liu B et al. *JAMA Network Open*.

46180	Tupelo, MS Micro Area	0.1038	0	0.016	0.202	0.044	0	0.716	183
13100	Beatrice, NE Micro Area	0.1039	0.017	0	0	0.032	0	0.916	57
14820	Brevard, NC Micro Area	0.1042	0	0	0.104	0.021	0	0.833	48
28580	Key West, FL Micro Area	0.1043	0.017	0.026	0.07	0.33	0	0.513	115
34620	Muncie, IN Metro Area	0.1047	0.016	0.005	0.052	0.021	0.005	0.869	191
36340	Oil City, PA Micro Area	0.1048	0.012	0	0	0.012	0	0.957	85
19180	Danville, IL Metro Area	0.1053	0	0	0.184	0.053	0.009	0.693	113
30700	Lincoln, NE Metro Area	0.1053	0.008	0.044	0.036	0.117	0	0.705	655
47780	Washington, IN Micro Area	0.1053	0	0	0	0.105	0	0.895	38
37900	Peoria, IL Metro Area	0.1057	0.006	0.027	0.111	0.049	0	0.76	662
27780	Johnstown, PA Metro Area	0.1065	0	0.005	0.051	0.009	0	0.884	215
22280	Fernley, NV Micro Area	0.1068	0.01	0.01	0	0.184	0.01	0.757	103
28300	Keene, NH Micro Area	0.1069	0.01	0.01	0	0.039	0	0.921	102
21220	Elko, NV Micro Area	0.1071	0.036	0	0.018	0.143	0	0.768	56
40760	Ruidoso, NM Micro Area	0.1071	0.179	0.071	0	0.321	0	0.429	28
10420	Akron, OH Metro Area	0.1076	0	0.017	0.134	0.023	0.002	0.783	1199
11700	Asheville, NC Metro Area	0.1078	0.004	0.009	0.065	0.101	0	0.767	696
33340	Milwaukee-Waukesha-West Allis, WI Metro Area	0.1085	0.004	0.035	0.204	0.153	0.001	0.571	2663
29100	La Crosse-Onalaska, WI-MN Metro Area	0.1086	0	0.042	0.043	0.027	0	0.832	322
32100	Marquette, MI Micro Area	0.1087	0.037	0	0.024	0.012	0	0.831	82
17820	Colorado Springs, CO Metro Area	0.1089	0.005	0.023	0.073	0.199	0.006	0.596	992
39940	Rexburg, ID Micro Area	0.1095	0	0.027	0.055	0.068	0	0.849	73
16580	Champaign-Urbana, IL Metro Area	0.1098	0	0.029	0.161	0.075	0.005	0.683	373
35580	New Ulm, MN Micro Area	0.1099	0.012	0	0.007	0.179	0	0.762	81
31940	Marinette, WI-MI Micro Area	0.1101	0.018	0	0.009	0.018	0	0.92	108
24300	Grand Junction, CO Metro Area	0.1105	0.005	0	0.005	0.12	0.014	0.805	217
37140	Paducah, KY-IL Micro Area	0.1108	0	0	0.102	0.043	0.009	0.778	117
47180	Vincennes, IN Micro Area	0.1111	0	0	0.022	0.022	0	0.844	45
14260	Boise City, ID Metro Area	0.1112	0.009	0.021	0.012	0.151	0	0.761	1249
10500	Albany, GA Metro Area	0.1118	0	0.013	0.539	0.013	0.013	0.395	152
18900	Crossville, TN Micro Area	0.1124	0	0	0.011	0.022	0.011	0.955	89
28100	Kankakee, IL Metro Area	0.1124	0	0	0.179	0.074	0	0.711	195
19860	Dickinson, ND Micro Area	0.1125	0.022	0	0.022	0.022	0	0.865	44
14180	Blytheville, AR Micro Area	0.1127	0	0	0.301	0.053	0	0.609	53
37660	Parsons, KS Micro Area	0.1127	0.033	0	0	0.113	0	0.703	26

© 2023 Liu B et al. *JAMA Network Open*.

10220	Ada, OK Micro Area	0.1136	0.323	0	0	0.076	0	0.488	52
29620	Lansing-East Lansing, MI Metro Area	0.1137	0.007	0.039	0.114	0.079	0	0.701	712
42220	Santa Rosa, CA Metro Area	0.1142	0.009	0.054	0.015	0.271	0.004	0.595	542
30860	Logan, UT-ID Metro Area	0.1145	0.011	0.017	0.006	0.12	0.011	0.812	174
28940	Knoxville, TN Metro Area	0.115	0.001	0.015	0.07	0.044	0.001	0.837	1357
34940	Naples-Immokalee-Marco Island, FL Metro Area	0.1152	0.003	0.012	0.112	0.485	0.003	0.355	330
35380	New Orleans-Metairie, LA Metro Area	0.1152	0.002	0.035	0.347	0.1	0	0.491	1241
39500	Quincy, IL-MO Micro Area	0.1154	0.008	0.008	0.031	0.008	0	0.892	129
43500	Silver City, NM Micro Area	0.1154	0	0	0	0.615	0	0.346	26
33740	Monroe, LA Metro Area	0.1158	0	0.026	0.432	0.021	0	0.505	190
39540	Racine, WI Metro Area	0.116	0.003	0.012	0.158	0.173	0	0.638	336
42300	Sault Ste. Marie, MI Micro Area	0.1167	0.3	0	0.033	0	0	0.65	60
32000	Marion, NC Micro Area	0.117	0	0.021	0.043	0.053	0	0.819	94
42020	San Luis Obispo-Paso Robles-Arroyo Grande, CA Metro Area	0.1172	0.014	0.041	0.011	0.262	0	0.614	443
29180	Lafayette, LA Metro Area	0.1174	0	0.027	0.348	0.017	0	0.581	477
47660	Warrensburg, MO Micro Area	0.1188	0	0	0.043	0.045	0	0.869	92
35020	Natchez, MS-LA Micro Area	0.119	0	0.024	0.595	0	0	0.381	42
17460	Cleveland-Elyria, OH Metro Area	0.1195	0.001	0.023	0.222	0.075	0.001	0.639	3649
47980	Watertown, SD Micro Area	0.1207	0.052	0	0.017	0.017	0	0.879	58
14020	Bloomington, IN Metro Area	0.121	0.003	0.019	0.037	0.031	0	0.859	322
28020	Kalamazoo-Portage, MI Metro Area	0.1217	0.006	0.006	0.109	0.075	0	0.751	345
16020	Cape Girardeau, MO-IL Metro Area	0.1218	0	0.012	0.122	0.012	0	0.817	164
28900	Klamath Falls, OR Micro Area	0.1228	0.018	0.018	0	0.053	0	0.825	57
48020	Watertown-Fort Atkinson, WI Micro Area	0.1228	0	0.01	0.004	0.121	0	0.845	122
49100	Winona, MN Micro Area	0.1232	0	0.037	0.039	0.014	0	0.869	97
48300	Wenatchee, WA Metro Area	0.1233	0.022	0	0	0.28	0	0.651	170
48820	Willmar, MN Micro Area	0.1245	0	0	0.058	0.141	0	0.801	120
43380	Sidney, OH Micro Area	0.1248	0	0.023	0.034	0.023	0	0.875	88
24460	Great Bend, KS Micro Area	0.125	0	0	0	0.167	0	0.792	24
46860	Vernal, UT Micro Area	0.125	0.05	0	0.025	0.075	0	0.85	40
30380	Lewistown, PA Micro Area	0.1266	0	0	0	0.013	0	0.945	78
41760	Sandpoint, ID Micro Area	0.1267	0	0	0	0.109	0	0.837	55
13460	Bend-Redmond, OR Metro Area	0.1276	0.011	0.023	0	0.084	0	0.853	266

© 2023 Liu B et al. *JAMA Network Open*.

30260	Lewisburg, PA Micro Area	0.1282	0	0	0	0.087	0	0.901	54
48220	Weatherford, OK Micro Area	0.1289	0.064	0	0.064	0.162	0	0.583	31
23540	Gainesville, FL Metro Area	0.1291	0.006	0.018	0.318	0.105	0	0.489	333
23900	Gettysburg, PA Metro Area	0.1294	0	0	0.035	0.165	0	0.788	85
35260	New Castle, PA Micro Area	0.1294	0	0.011	0.036	0.012	0	0.916	162
41460	Salina, KS Micro Area	0.1298	0	0.009	0.019	0.121	0	0.768	107
15380	Buffalo-Cheektowaga-Niagara Falls, NY Metro Area	0.1299	0.003	0.031	0.124	0.092	0.001	0.71	1016
43700	Somerset, KY Micro Area	0.1299	0	0.013	0	0.026	0	0.961	77
27060	Ithaca, NY Metro Area	0.13	0.001	0.059	0.078	0.069	0	0.752	99
12900	Batesville, AR Micro Area	0.1303	0	0	0.016	0.049	0	0.902	61
45220	Tallahassee, FL Metro Area	0.1303	0.004	0.029	0.385	0.042	0.002	0.501	545
13500	Bennettsville, SC Micro Area	0.1304	0	0	0.739	0	0	0.174	23
19000	Cullowhee, NC Micro Area	0.1304	0.065	0	0.022	0.065	0	0.804	46
26940	Indianola, MS Micro Area	0.1304	0	0	0.913	0.043	0	0.043	23
22300	Findlay, OH Micro Area	0.1313	0	0.019	0	0.089	0	0.835	159
41780	Sandusky, OH Micro Area	0.1317	0	0	0.076	0.045	0	0.813	144
27300	Jacksonville, IL Micro Area	0.1322	0	0	0.036	0.048	0	0.891	83
13780	Binghamton, NY Metro Area	0.1324	0.004	0.033	0.093	0.052	0	0.776	279
32540	McAlester, OK Micro Area	0.1324	0.471	0	0	0.029	0	0.441	68
23380	Fremont, OH Micro Area	0.1325	0	0	0.039	0.113	0	0.829	90
26260	Hope, AR Micro Area	0.1329	0	0	0.331	0.11	0	0.536	45
20500	Durham-Chapel Hill, NC Metro Area	0.1338	0.003	0.042	0.358	0.154	0	0.394	897
35620	New York-Newark-Jersey City, NY-NJ-PA Metro Area	0.1358	0.003	0.099	0.137	0.28	0.003	0.46	22099
43220	Shelton, WA Micro Area	0.1361	0.036	0.009	0.015	0.136	0	0.735	110
18500	Corning, NY Micro Area	0.1373	0	0.014	0.012	0	0	0.951	131
20100	Dover, DE Metro Area	0.1376	0.01	0.031	0.283	0.095	0	0.531	297
12700	Barnstable Town, MA Metro Area	0.1388	0.006	0.016	0.044	0.044	0	0.845	317
29780	Las Vegas, NM Micro Area	0.1389	0	0	0.028	0.833	0	0.139	36
48580	Whitewater-Elkhorn, WI Micro Area	0.1392	0.005	0.012	0.005	0.172	0	0.765	186
19740	Denver-Aurora-Lakewood, CO Metro Area	0.1395	0.008	0.035	0.056	0.263	0.002	0.586	3275
12580	Baltimore-Columbia-Towson, MD Metro Area	0.1403	0.001	0.062	0.414	0.068	0.001	0.408	4177
22860	Fort Polk South, LA Micro Area	0.1404	0	0	0.175	0.07	0	0.684	57
27020	Iron Mountain, MI-WI Micro Area	0.1407	0	0	0	0.023	0	0.953	42

© 2023 Liu B et al. *JAMA Network Open*.

40300	Rochelle, IL Micro Area	0.1412	0	0	0.009	0.185	0	0.797	84
22380	Flagstaff, AZ Metro Area	0.1422	0.307	0	0	0.158	0	0.472	133
23300	Freeport, IL Micro Area	0.1426	0	0.008	0.228	0.053	0.014	0.578	70
15700	Cambridge, MD Micro Area	0.1429	0	0.016	0.333	0.079	0	0.54	63
47460	Walla Walla, WA Metro Area	0.1429	0	0.013	0	0.312	0	0.636	77
27460	Jamestown-Dunkirk-Fredonia, NY Micro Area	0.1437	0.008	0	0.008	0.158	0	0.766	132
30780	Little Rock-North Little Rock-Conway, AR Metro Area	0.1438	0.003	0.017	0.269	0.062	0	0.632	1078
32700	McPherson, KS Micro Area	0.1453	0	0	0	0.074	0	0.926	48
28500	Kerrville, TX Micro Area	0.1454	0	0	0.036	0.415	0	0.513	55
28740	Kingston, NY Metro Area	0.1454	0	0.012	0.031	0.111	0	0.818	261
36620	Ontario, OR-ID Micro Area	0.1466	0	0	0	0.319	0	0.647	116
31620	Magnolia, AR Micro Area	0.1467	0	0.049	0.496	0.101	0	0.354	20
38700	Pontiac, IL Micro Area	0.148	0.009	0	0.031	0.057	0	0.833	87
35980	Norwich-New London, CT Metro Area	0.1483	0.006	0.048	0.057	0.19	0.002	0.641	526
38420	Platteville, WI Micro Area	0.1487	0	0.023	0.015	0	0	0.929	67
48940	Wilmington, OH Micro Area	0.1496	0	0	0.025	0.012	0	0.926	80
10140	Aberdeen, WA Micro Area	0.1507	0.032	0.008	0.002	0.11	0	0.766	126
46020	Truckee-Grass Valley, CA Micro Area	0.1516	0.01	0.01	0.02	0.105	0.01	0.825	98
13180	Beaver Dam, WI Micro Area	0.1519	0	0.008	0.028	0.08	0	0.864	177
24330	Grand Rapids, MN Micro Area	0.1522	0.038	0	0.013	0.025	0	0.888	78
45140	Tahlequah, OK Micro Area	0.1526	0.483	0	0.021	0.056	0	0.316	104
45780	Toledo, OH Metro Area	0.1527	0.003	0.024	0.138	0.071	0	0.716	1146
37540	Paris, TN Micro Area	0.1538	0	0	0.077	0.077	0	0.692	13
34300	Mountain Home, ID Micro Area	0.1539	0	0.044	0	0.15	0	0.74	45
17860	Columbia, MO Metro Area	0.1559	0.004	0.033	0.123	0.065	0.007	0.706	275
12100	Atlantic City-Hammonton, NJ Metro Area	0.1561	0	0.124	0.135	0.233	0	0.468	378
34060	Morgantown, WV Metro Area	0.157	0	0.008	0.033	0.033	0	0.893	121
11260	Anchorage, AK Metro Area	0.1573	0.09	0.067	0.028	0.082	0.024	0.567	744
36140	Ocean City, NJ Metro Area	0.1573	0	0.011	0.067	0.146	0	0.775	89
12420	Austin-Round Rock, TX Metro Area	0.1574	0.003	0.066	0.085	0.348	0	0.455	4002
25540	Hartford-West Hartford-East Hartford, CT Metro Area	0.1581	0.001	0.057	0.125	0.203	0	0.58	2106
25940	Hilton Head Island-Bluffton-Beaufort, SC Metro Area	0.1583	0	0.008	0.317	0.25	0	0.367	120

© 2023 Liu B et al. *JAMA Network Open*.

33860	Montgomery, AL Metro Area	0.1587	0	0.01	0.583	0.02	0	0.359	497
11820	Astoria, OR Micro Area	0.1592	0.016	0	0.016	0.128	0	0.836	62
44100	Springfield, IL Metro Area	0.161	0.002	0.032	0.137	0.026	0.002	0.74	422
35300	New Haven-Milford, CT Metro Area	0.1617	0.003	0.048	0.158	0.23	0	0.529	1330
29340	Lake Charles, LA Metro Area	0.162	0.009	0.014	0.343	0.046	0	0.579	216
21540	Escanaba, MI Micro Area	0.1626	0.032	0	0	0	0	0.968	30
17700	Coffeyville, KS Micro Area	0.1629	0.023	0	0.07	0.023	0	0.744	42
42940	Sevierville, TN Micro Area	0.1638	0	0.017	0.009	0.043	0	0.931	116
20780	Edwards, CO Micro Area	0.164	0.02	0.02	0.005	0.473	0	0.439	36
43620	Sioux Falls, SD Metro Area	0.1642	0.026	0.014	0.067	0.072	0	0.797	420
12940	Baton Rouge, LA Metro Area	0.1648	0.001	0.022	0.444	0.045	0.001	0.477	728
42100	Santa Cruz-Watsonville, CA Metro Area	0.1665	0.003	0.068	0.014	0.396	0	0.492	300
20420	Durango, CO Micro Area	0.1667	0.071	0	0	0.143	0	0.714	42
39220	Price, UT Micro Area	0.1667	0	0	0	0.167	0	0.833	18
48500	West Point, MS Micro Area	0.1667	0	0	0.75	0	0	0.25	24
49460	Yankton, SD Micro Area	0.1685	0.024	0.024	0	0.048	0	0.904	41
46540	Utica-Rome, NY Metro Area	0.1695	0.001	0.015	0.069	0.059	0	0.841	277
10580	Albany-Schenectady-Troy, NY Metro Area	0.1699	0.005	0.046	0.12	0.1	0	0.691	1235
21420	Enid, OK Metro Area	0.1724	0.046	0	0.011	0.092	0	0.816	87
12780	Bartlesville, OK Micro Area	0.1749	0.185	0	0.016	0.099	0	0.676	51
19060	Cumberland, MD-WV Metro Area	0.1753	0	0.01	0.041	0.01	0	0.918	97
37740	Payson, AZ Micro Area	0.1759	0.135	0	0	0.191	0	0.66	68
38780	Portales, NM Micro Area	0.1768	0	0	0	0.279	0	0.721	22
46060	Tucson, AZ Metro Area	0.1771	0.027	0.031	0.039	0.404	0.001	0.454	1276
13740	Billings, MT Metro Area	0.1774	0.065	0	0.024	0.097	0.008	0.718	124
10100	Aberdeen, SD Micro Area	0.1776	0.075	0	0.009	0.037	0	0.86	107
11620	Ardmore, OK Micro Area	0.1781	0.11	0	0.068	0.055	0	0.685	73
35740	Norfolk, NE Micro Area	0.1784	0	0	0.049	0.162	0	0.784	61
13720	Big Stone Gap, VA Micro Area	0.1803	0	0	0.033	0.016	0	0.934	61
29300	LaGrange, GA Micro Area	0.1803	0	0.033	0.361	0.016	0	0.557	61
31680	Malvern, AR Micro Area	0.1806	0	0	0.063	0.114	0	0.754	60
32140	Marshall, MN Micro Area	0.1807	0	0.036	0.024	0.048	0	0.855	83
11060	Altus, OK Micro Area	0.1812	0	0	0.045	0.455	0	0.455	22
30280	Lewisburg, TN Micro Area	0.1818	0	0	0.114	0.091	0	0.773	44

© 2023 Liu B et al. *JAMA Network Open*.

39420	Pullman, WA Micro Area	0.1821	0.003	0.043	0.003	0.165	0	0.726	49
12020	Athens-Clarke County, GA Metro Area	0.1834	0	0.017	0.292	0.112	0	0.544	349
26740	Hutchinson, KS Micro Area	0.1834	0.011	0	0.024	0.126	0	0.773	87
33580	Mitchell, SD Micro Area	0.1836	0.052	0.026	0.026	0.052	0	0.816	38
24220	Grand Forks, ND-MN Metro Area	0.184	0.061	0.009	0.014	0.061	0.005	0.835	212
45900	Traverse City, MI Micro Area	0.1856	0.015	0.007	0	0.022	0	0.904	134
43740	Somerset, PA Micro Area	0.1865	0	0.012	0.012	0.012	0	0.953	85
11860	Atchison, KS Micro Area	0.1893	0.262	0	0.076	0.032	0	0.588	26
20460	Durant, OK Micro Area	0.1897	0.259	0	0.052	0	0	0.638	58
13620	Berlin, NH-VT Micro Area	0.1915	0.021	0.021	0	0	0	0.915	47
15060	Brookings, OR Micro Area	0.1923	0.038	0	0	0.077	0	0.769	26
38540	Pocatello, ID Metro Area	0.1926	0.102	0.011	0.011	0.137	0	0.738	88
19940	Dixon, IL Micro Area	0.193	0	0	0.019	0.147	0.005	0.792	62
15860	CaÃ±on City, CO Micro Area	0.1934	0	0	0	0.048	0	0.836	41
31220	Ludington, MI Micro Area	0.1951	0	0.024	0	0.024	0	0.902	41
25700	Hays, KS Micro Area	0.1957	0	0	0	0.065	0	0.87	46
12860	Batavia, NY Micro Area	0.1984	0	0.028	0.042	0.029	0	0.869	70
14860	Bridgeport-Stamford-Norwalk, CT Metro Area	0.1994	0.003	0.061	0.136	0.21	0	0.567	1570
26700	Huron, SD Micro Area	0.2	0	0	0.029	0.171	0	0.8	35
33940	Montrose, CO Micro Area	0.2008	0.015	0	0	0.217	0	0.729	64
28060	Kalispell, MT Micro Area	0.2043	0.011	0.032	0.022	0.011	0.022	0.839	93
14580	Bozeman, MT Micro Area	0.2046	0.029	0	0.015	0.117	0	0.766	68
19340	Davenport-Moline-Rock Island, IA-IL Metro Area	0.2048	0.009	0.009	0.079	0.119	0	0.741	214
22660	Fort Collins, CO Metro Area	0.2049	0.006	0.032	0.02	0.168	0.003	0.752	385
23240	Fredericksburg, TX Micro Area	0.2065	0	0	0	0.452	0	0.548	33
16060	Carbondale-Marion, IL Metro Area	0.2077	0	0.015	0.096	0.015	0	0.838	197
27940	Juneau, AK Micro Area	0.2113	0.127	0.085	0.014	0.028	0.014	0.521	71
30580	Liberal, KS Micro Area	0.2143	0	0.107	0	0.571	0	0.321	28
29660	Laramie, WY Micro Area	0.2162	0	0.054	0	0.162	0	0.703	37
16620	Charleston, WV Metro Area	0.2167	0	0.011	0.111	0.006	0	0.85	180
44660	Stillwater, OK Micro Area	0.2171	0.096	0.014	0.056	0.05	0	0.709	73
45860	Torrington, CT Micro Area	0.2182	0	0.018	0.012	0.101	0.003	0.843	325
16940	Cheyenne, WY Metro Area	0.2185	0.013	0.007	0.007	0.079	0	0.801	151
38620	Ponca City, OK Micro Area	0.2218	0.109	0	0	0.052	0.017	0.734	40

© 2023 Liu B et al. *JAMA Network Open*.

11740	Ashland, OH Micro Area	0.2221	0	0.031	0.004	0.016	0	0.925	63
37500	Paragould, AR Micro Area	0.2291	0	0	0.07	0.017	0	0.912	56
43260	Sheridan, WY Micro Area	0.2292	0	0	0	0	0	0.958	48
45020	Sweetwater, TX Micro Area	0.2302	0	0	0	0.307	0	0.693	13
36830	Othello, WA Micro Area	0.2317	0	0	0	0.709	0	0.287	17
36840	Ottawa, KS Micro Area	0.2334	0	0	0.03	0.106	0	0.862	34
30660	Lincoln, IL Micro Area	0.234	0	0	0.047	0	0	0.88	42
32860	Menomonie, WI Micro Area	0.236	0	0.014	0.02	0.014	0.009	0.902	105
36300	Ogdensburg-Massena, NY Micro Area	0.2374	0.048	0	0	0.018	0	0.924	109
18180	Concord, NH Micro Area	0.2376	0.004	0.019	0.011	0.036	0.002	0.916	260
21180	Elkins, WV Micro Area	0.2381	0	0	0	0	0	1	21
14500	Boulder, CO Metro Area	0.2399	0.004	0.055	0.004	0.154	0.001	0.748	483
36580	Oneonta, NY Micro Area	0.2425	0	0.027	0.014	0.081	0	0.851	74
19580	Defiance, OH Micro Area	0.2447	0	0	0.021	0.085	0	0.883	94
22800	Fort Madison-Keokuk, IA-IL-MO Micro Area	0.2448	0	0	0	0	0	0.922	36
33540	Missoula, MT Metro Area	0.2452	0.071	0.019	0.013	0.039	0	0.832	155
33500	Minot, ND Micro Area	0.2456	0.035	0	0.064	0.064	0.006	0.789	171
45820	Topeka, KS Metro Area	0.249	0.009	0.006	0.066	0.14	0.003	0.656	333
27620	Jefferson City, MO Metro Area	0.2493	0.004	0.007	0.087	0.025	0	0.841	276
34500	Mount Vernon, IL Micro Area	0.2504	0	0	0.016	0	0	0.937	63
38340	Pittsfield, MA Metro Area	0.2533	0	0.01	0.034	0.058	0	0.884	205
12180	Auburn, NY Micro Area	0.258	0	0	0.035	0.003	0.007	0.87	85
12300	Augusta-Waterville, ME Micro Area	0.2595	0.005	0	0.035	0.025	0	0.918	208
34660	Murray, KY Micro Area	0.2619	0	0.024	0.024	0.048	0	0.857	42
42900	Seneca Falls, NY Micro Area	0.2623	0.044	0.001	0.058	0.003	0	0.85	22
45340	Taos, NM Micro Area	0.2641	0.038	0	0	0.578	0.038	0.346	26
43580	Sioux City, IA-NE-SD Metro Area	0.2654	0.035	0.017	0.035	0.196	0	0.716	60
40180	Riverton, WY Micro Area	0.2667	0.4	0	0.022	0.044	0	0.467	45
45940	Trenton, NJ Metro Area	0.2698	0.003	0.181	0.164	0.215	0	0.411	626
25720	Heber, UT Micro Area	0.2703	0	0	0	0.054	0	0.946	37
28540	Ketchikan, AK Micro Area	0.2778	0.333	0.111	0	0	0	0.556	18
19500	Decatur, IL Metro Area	0.2826	0	0	0.153	0.058	0	0.674	84
21380	Emporia, KS Micro Area	0.2826	0	0.019	0.019	0.151	0	0.774	53
25100	Guymon, OK Micro Area	0.2857	0	0	0	0.357	0	0.571	14

© 2023 Liu B et al. *JAMA Network Open*.

13900	Bismarck, ND Metro Area	0.2864	0.069	0.005	0.016	0.069	0	0.814	188
46820	Vermillion, SD Micro Area	0.2869	0.064	0	0.064	0.064	0	0.742	13
24060	Glenwood Springs, CO Micro Area	0.2931	0.004	0.004	0.013	0.403	0	0.522	61
23780	Garden City, KS Micro Area	0.2954	0	0.025	0.025	0.64	0	0.271	40
16460	Centralia, IL Micro Area	0.2995	0	0	0.018	0.017	0	0.9	56
32340	Maryville, MO Micro Area	0.3	0	0	0	0	0	1	20
37940	Peru, IN Micro Area	0.3083	0	0	0	0.039	0	0.958	51
17740	Coldwater, MI Micro Area	0.3141	0.004	0	0	0.084	0	0.912	22
26460	Hudson, NY Micro Area	0.3143	0	0.038	0.063	0.068	0	0.779	79
16660	Charleston-Mattoon, IL Micro Area	0.3185	0	0.011	0.032	0	0	0.944	94
42140	Santa Fe, NM Metro Area	0.3232	0.062	0.016	0.031	0.6	0	0.283	129
34140	Moscow, ID Micro Area	0.3239	0.022	0.086	0	0.058	0	0.82	46
24100	Gloversville, NY Micro Area	0.3327	0	0	0.001	0.006	0	0.963	72
13940	Blackfoot, ID Micro Area	0.3399	0.055	0.002	0.003	0.158	0	0.772	52
31380	Macomb, IL Micro Area	0.3505	0	0.044	0.044	0.044	0	0.786	45
20660	Easton, MD Micro Area	0.3529	0	0.029	0.206	0.029	0	0.706	34
44540	Sterling, CO Micro Area	0.3612	0.08	0	0	0.04	0.04	0.759	24
23820	Gardnerville Ranchos, NV Micro Area	0.3729	0.034	0.034	0	0.153	0	0.695	59
25740	Helena, MT Micro Area	0.3885	0.01	0	0	0.052	0	0.916	95
49260	Woodward, OK Micro Area	0.3913	0	0	0	0.174	0	0.826	23
36460	Olean, NY Micro Area	0.3973	0	0	0	0.041	0	0.957	50
14720	Breckenridge, CO Micro Area	0.4	0	0	0	0.1	0	0.9	10
15580	Butte-Silver Bow, MT Micro Area	0.4681	0.028	0.055	0.055	0.028	0	0.835	36
47420	Wahpeton, ND-MN Micro Area	0.4859	0.042	0	0	0.067	0	0.859	43
27420	Jamestown, ND Micro Area	0.5405	0	0	0.054	0.027	0	0.892	37
24500	Great Falls, MT Metro Area	0.6486	0.027	0	0.054	0.135	0	0.703	37
44460	Steamboat Springs, CO Micro Area	0.6522	0.002	0.002	0	0.129	0	0.864	23
15460	Burlington, IA-IL Micro Area	0.8129	0	0	0	0.065	0	0.903	6
46520	Urban Honolulu, HI Metro Area	0.9067	0.002	0.283	0.019	0.144	0.192	0.205	144

© 2023 Liu B et al. *JAMA Network Open*.
