# Supplementary material for: Racial and Ethnic Disparities in Geographic Access to Autism Resources Across the US
Source: JAMA Netw Open. 2023 Jan 23;6(1):e2251182. doi: 10.1001/jamanetworkopen.2022.51182 (PMC9871799; doi:10.1001/jamanetworkopen.2022.51182)
Supplement: Supplement 2. — Data Sharing Statement [file jamanetwopen-e2251182-s002.pdf]

Data Sharing Statement

Liu. Racial and Ethnic Disparities in Geographic Access to Autism Resources Across the US. *JAMA Netw Open*. Published January 23, 2023. doi:10.1001/jamanetworkopen.2022.51182

Data
Data available: Yes
Data types: Deidentified participant data
How to access data: data related to resources by geography defined as CBSA is available in the supplementary materials. The complete set of autism healthcare resources is available at gapmap.stanford.edu
When available: With publication

Supporting Documents
Document types: None

Additional Information
Who can access the data: data will be available openly
Types of analyses: all code used to run the analysis will be made available by request
Mechanisms of data availability: data used in the manuscript will be open sourced.
